# Spatial distribution of lamin A/C determines nuclear stiffness and stress-mediated deformation

**DOI:** 10.1242/jcs.248559

**Published:** 2021-05-24

**Authors:** Luv Kishore Srivastava, Zhaoping Ju, Ajinkya Ghagre, Allen J. Ehrlicher

**Affiliations:** 1 Department of Bioengineering, McGill University, Montreal H3A 0E9; 2 Department of Anatomy and Cell Biology, McGill University, Montreal H3A 0C7, Canada; 3 Department of Biomedical Engineering, McGill University, Montreal H3A 2B4, Canada; 4 Department of Mechanical Engineering, McGill University, Montreal H3A 0C3; 5 Centre for Structural Biology, McGill University, Montreal H3G 0B1, Canada; 6 Goodman Cancer Research Centre, McGill University, Montreal H3A 1A3, Canada

**Keywords:** Bulk moduli, Heterogeneity, Lamin A/C, Mechanotransduction

## Abstract

While diverse cellular components have been identified as mechanotransduction elements, the deformation of the nucleus itself is a critical mechanosensory mechanism, implying that nuclear stiffness is essential in determining responses to intracellular and extracellular stresses. Although the nuclear membrane protein lamin A/C is known to contribute to nuclear stiffness, bulk moduli of nuclei have not been reported for various levels of lamin A/C. Here, we measure the nuclear bulk moduli as a function of lamin A/C expression and applied osmotic stress, revealing a linear dependence within the range of 2–4 MPa. We also find that the nuclear compression is anisotropic, with the vertical axis of the nucleus being more compliant than the minor and major axes in the substrate plane. We then related the spatial distribution of lamin A/C with submicron 3D nuclear envelope deformation, revealing that local areas of the nuclear envelope with higher density of lamin A/C have correspondingly lower local deformations. These findings describe the complex dispersion of nuclear deformations as a function of lamin A/C expression and distribution, implicating a lamin A/C role in mechanotransduction.

This article has an associated First Person interview with the first author of the paper.

## Abstract (precis)

**Summary:** The nuclear scaffold protein lamin A/C modulates nuclear stiffness, and heterogeneous lamin A/C density leads to diverse nuclear strain under stress.

## INTRODUCTION

The nucleus is one of the most crucial organelles and the storehouse of DNA in the cell, integrating diverse biochemical cues for proper cell function (Lamond and Earnshaw, 1998). In addition to biochemical cues, the nucleus has an increasingly clear role as a mechanosensory structure in the cell (Kirby and Lammerding, 2018). As a mechanosensor, the stiffness of the nucleus determines specific deformations in response to applied stresses. The nucleus is notably stiffer than the cytoplasm (0.5–3 kPa) (Chen et al., 2012), with an effective Young's modulus ranging from 1–10 kPa (Caille et al., 2002; Vaziri and Mofrad, 2007); while these values appear relatively consistent in a given cell line (Khavari and Ehrlicher, 2019), multiple factors may cause nuclear stiffness to change significantly. For example, the stiffness of nuclei varies 5-fold during cell division (Chu et al., 2017), in stem cells the stiffness can increase 6-fold over the differentiation process (Pajerowski et al., 2007), and in many types of cancers the structure of the nucleus is altered and the stiffness is vastly reduced (Lin et al., 2015). Many of these morphological and mechanical changes are the result of the changes in nuclear envelope architecture (Bell and Lammerding, 2016).

The nuclear envelope is composed of double-membraned bi-lipid layers with a 30–50 nm perinuclear space between the membranes. The inner nuclear membrane is held in place by a consortium of more than 50 membrane proteins. Some of the key proteins present in the inner nuclear membrane include emerin, SUN1, SUN2, lamins and lamin-associated proteins (Hetzer, 2010). Lamins are type V intermediate filaments mainly localized under the nuclear membrane with a small fraction present in nucleoplasm. The inner membrane is held in place by the underlying nuclear lamina, which is mainly composed of A/C type (encoded by one gene, *LMNA*) and B type lamins. Lamin A/C has a profound effect on the stiffness of the nucleus, but lamin B shows very little or no effect on the nuclear stiffness, thus making lamin A/C a key component in nuclear mechanics (Dechat et al., 2010). Besides this, it has previously been shown that nuclear membrane-localized lamin A/C is major contributor to nuclear mechanics compared to the fraction present in the nucleoplasm making it a critical factor in studying nuclear mechanotransduction (Zwerger et al., 2015).

Numerous studies have illustrated the significance of lamin A/C in determining the stiffness of the nucleus. Suppression of lamin A/C increases nuclear compliance, and nuclei in lamin A/C-knockout cells display 30% to 50% more deformation than wild-type (WT) cells when probed with substrate stretch or magnetic microrheology, respectively (Lammerding et al., 2004). Previous studies have also shown that nuclei in lamin A/C-knockout cells are more fragile, with a greater susceptibility to rupture under increased intranuclear pressure (Hanson et al., 2015). Nuclei isolated from cells transfected with shRNA for lamin A/C showed 1.5–1.7 times more bead displacement compared to WT nuclei in magnetic bead microrheology, indicating the role of lamin A/C in determining the stiffness of the nucleus (Guilluy et al., 2014). Conversely, overexpression of lamin A/C stiffens nuclei; when 3T3 fibroblasts are transfected with lamin A/C and seeded on vertical nanopillars, they showed 40–50% less nanopillar-induced deformation in both intact and isolated nuclei (Hanson et al., 2015). Lamin A/C expression and nuclear stiffness also directly affect the behavior of the cell; as an example, lamin A/C overexpression hinders 3D cell migration through micropores (Harada et al., 2014); however, it seems to facilitate 2D migration, as WT cells are marginally faster than lamin A/C-knockout cells (Wang et al., 2019). While lamin A/C expression clearly influences nuclear mechanics and cell behavior, the vast majority of these studies have looked at qualitative treatments of lamin A/C, that is, parsing cells into groups of overexpression, WT or knockdown. Harada et al. (2014) showed that in cancerous solid tissue cells and mesenchymal stem cells, the apparent nuclear stiffness increases with relative increasing of the ratio of lamin A to lamin B with a power law coefficient of 0.5, where lamin B levels vary little, suggesting that specifically lamin A expression levels play a key role in the nuclear elastic moduli. However, the quantitative scaling of nuclear bulk modulus as a function of lamin A/C expression remains unknown.

The expression of lamin A/C is highly variable, and changes during different stages of cell cycle (Moir et al., 2000), during stem cell differentiation (Schirmer and Gerace, 2004) and as a function of microenvironment stiffness (Crisp et al., 2006). In addition to overall expression levels, the distribution of lamin A/C appears heterogeneous, with the formation of foci and honeycomb patterns observed in the nuclear envelope (Barateau et al., 2017). Some studies have also indicated the nuclear deformation to be anisotropic (Tremblay et al., 2013; Haase et al., 2016), but it remains unknown how the spatial heterogeneity of lamin A/C affects nuclear deformation. Studies to date have focused on comparing phenotypical nuclear stiffness changes in ensemble population lamin A/C expression levels via overexpression or suppression (Lammerding et al., 2006), but have not examined the quantitative dependence of nuclear stiffness on lamin A/C expression, or the impact of the spatial distribution of lamin A/C on deformation.

To understand the quantitative impact of lamin A/C structure on nuclear mechanics, here, we measure the deformability and bulk moduli of nuclei as a function of lamin A/C expression. We identify a mechanical anisotropy of bulk compression, leading to increasing relative compliance in the major, minor, and *z*-axes, respectively. At the submicron level, we quantify the spatial distribution of lamin A/C and show that dense lamin A/C structures attenuate local deformation, providing heterogeneous nuclear deformations even under uniform nuclear stresses.

## RESULTS AND DISCUSSION

### Lamin A/C stiffens the nucleus

**Fig. 1.**
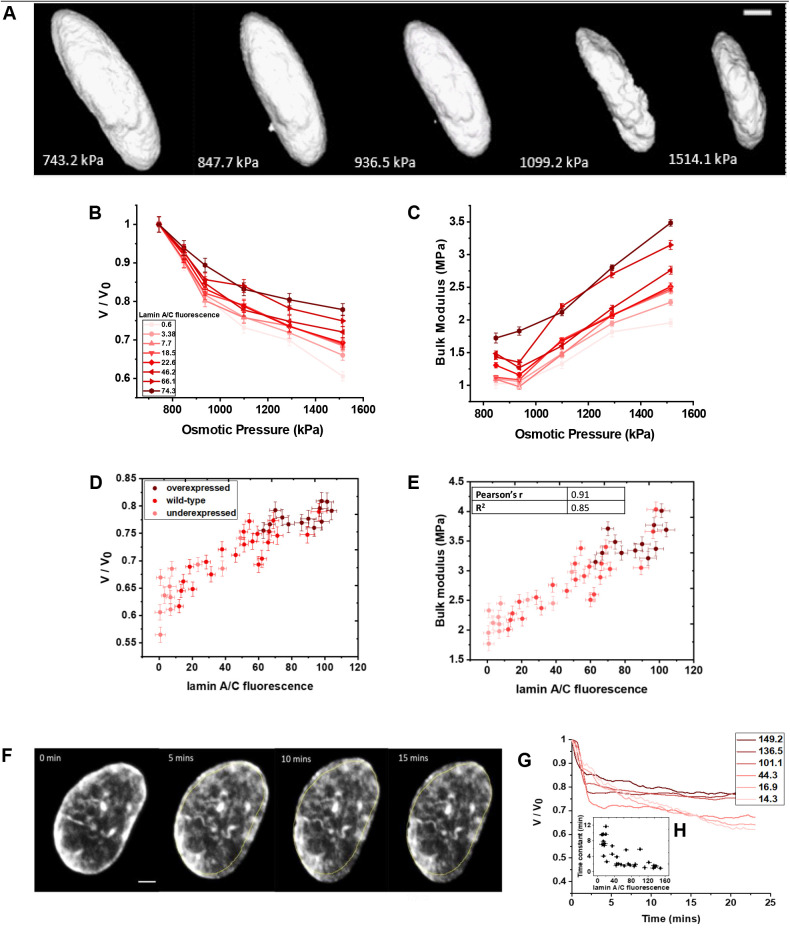
**Hyperosmotic compression of the nucleus in 3T3 Fibroblast cells.** (A) 3D reconstruction of an example nucleus within a cell at various osmotic pressures. Scale bar: 3 µm. (B) Normalized nuclear volumes of 3T3 cells transfected with lamin A/C chromobody showing that stiffness increases as the protein expression increases (fluorescence values in arbitrary units) (*n*=8). (C) Bulk moduli of 3T3 cells transfected with lamin A/C chromobody for showing that stiffness increases as the protein expression increases (*n*=8). (D) *V*/*V*_0_ increasing as a function of lamin A/C fluorescence under 1514.1 kPa osmotic pressure (*n*=46). (E) Nuclear stiffness in terms of bulk moduli increasing as a function of lamin A/C fluorescence under 1514.1 kPa osmotic pressure (*n*=46). (F) Example 3T3 fibroblast nuclei showing change in nuclear volume with time after PEG addition. Scale bar: 2 µm. (G) Change in nuclear volume as a function of time under 1514.1 kPa osmotic pressure as a function of lamin A/C fluorescence. (H) The time constant as a function of lamin A/C fluorescence (*n*=32). In D–F, the figures show data from three independent experiments. Quantitative data are mean±s.e.m.

To measure the bulk compressibility of the nuclei, we exposed nuclei with varying lamin A/C expression to different osmotic pressures using 400 Da polyethylene glycol (PEG). Since PEG is impermeable across the cell membrane, the hyperosmotic environment results in nuclear compression (Fig. 1A). This compression is due to the expulsion of water as the cell and nuclei regain their original volume when returned to isotonic medium (Guo et al., 2017; Khavari and Ehrlicher, 2019). Previous studies have shown that, under external osmotic pressure, nuclear volume changes along with the concentration of intracellular macromolecules, thus exerting compressive forces on it (Guo et al., 2017; Khavari and Ehrlicher, 2019). The pressure–volume curve for nuclei of WT NIH 3T3 fibroblast cells at different PEG 400 concentrations exerting different osmotic pressures shows that the nuclear compression was higher in cells expressing low levels of lamin A/C and vice versa (Fig. 1B).

This result is also reflected in the bulk moduli calculated from the change in the nuclear volume where the cells expressing more lamin A/C are relatively stiffer (Fig. 1C). A linear relationship could be seen when we plotted *V*/*V*_0_ (where *V*_0_ is original volume) and bulk moduli as a function of lamin A/C fluorescence, clearly indicating a direct relationship between lamin A/C expression and nuclear rigidity (Fig. 1D,E) with the bulk modulus for the highest lamin A/C-expressing cell at the highest exerted osmotic pressure being 4.01 Mpa. The slope calculated from Fig. 1E was found to be 0.01, which means that the nucleus stiffens by 1% with each unit increase in lamin A/C fluorescence. We also observed the nuclear volume as a function of time for different lamin A/C-expressing cells after PEG addition showing apparent regimes of gradual or rapid compression based on low or high lamin A/C expression, respectively. We noted that single exponential fits distinguish between the regimes, and plot six examples of this in Fig. 1G. We also report the compression time constant as a function of lamin A/C expression level, showing that with increasing expression, the time constant generally decreases (Fig. 1H).

### Nuclear stiffness is anisotropic

**Fig. 2.**
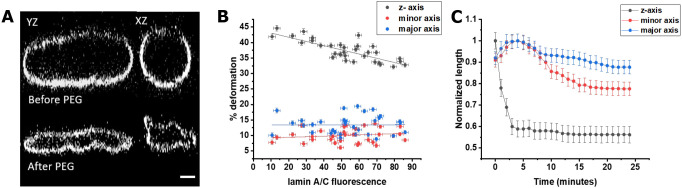
**Anisotropy in nuclear compression under PEG.** (A) Example of a WT nuclei in *y-z* and *x-z* plane before PEG addition (top) and after PEG addition (bottom) showing a large deformation in the *z*-axis. Scale bar: 3 µm. (B) Nuclear deformation percentage along the major, minor and *z*-axis in WT nuclei for different lamin A/C expression levels (*n*=44; data from three independent experiments) showing higher deformation along z-axis compared to the major and minor axis. (C) Example of a 3T3 fibroblast nucleus showing that the *z*-axis gets compressed instantly after PEG addition whereas major and minor axes expand in the beginning followed by reduction in the length showing anisotropy in nuclear compression with time. Quantitative data are mean±s.e.m. from three replicate measurements from one example experiment.

To measure the anisotropic nuclear deformation, we exposed the cells transfected with lamin A/C chromobody to PEG and imaged with confocal microscopy (Leica SP8) after 25 min of PEG addition. Our results reveal strong anisotropic nuclear deformation where the deformation in the *z*-axis was highest, followed by deformation  in the minor and major axis (Fig. 2A). When the percentage deformation was plotted, the *z*-axis was found to deform three to four times more in comparison to the minor and major axis, respectively (Fig. 2B; Fig. S2). Also, the deformation in *z*-axis was found to be more for cells expressing lower levels of lamin A/C and less cells expressing higher levels of lamin A/C, while the major and minor axes deformations showed a weaker relationship with lamin A/C concentration. To examine the dynamics of nuclear deformation under stress, we imaged the nuclei every 60 s after PEG addition. Here, the nucleus quickly flattens in the *z*-axis, resulting in a transient expansion along the major and minor axes, which starts to contract after 5 min (Fig. 2C). This shows a time dependency to nuclear compression after PEG addition.

### Lamin A/C density is spatially heterogeneous and leads to a variation in local compliance

**Fig. 3.**
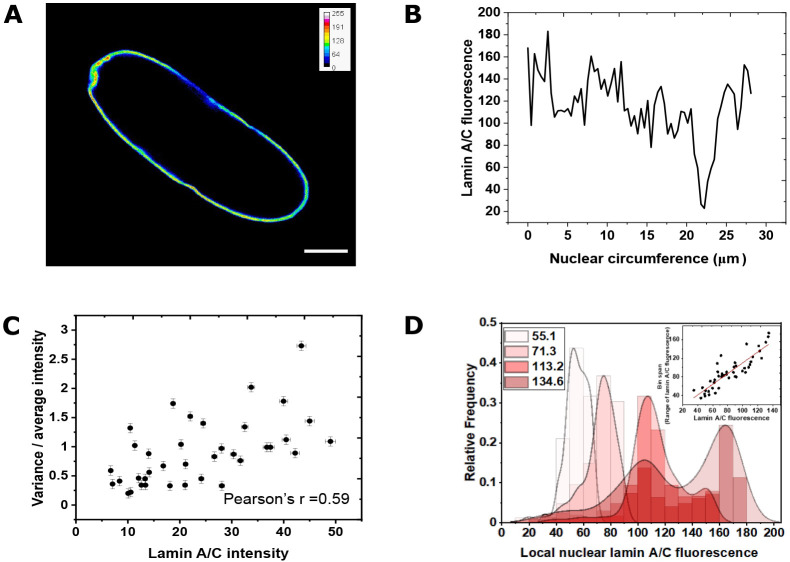
**Lamin A/C is spatially heterogeneous in the nucleus.** (A) Example 3T3 fibroblast cells transfected with lamin A chromobody showing variability in the spatial distribution along a cross-section nuclear membrane. Scale bar: 5 µm. (B) Quantification of lamin A/C heterogeneity along the nuclear membrane circumference shown in A. (C) Normalized variance of lamin A/C chromobody fluorescence as a function of total lamin A/C expression in different nuclei shows that higher expression of lamin A/C increases the heterogeneity of distribution (*n*=39; data from two independent experiments). Data are mean±s.e.m. (D) The histogram gets wider with increasing total lamin A/C fluorescence showing a wider range of local lamin A/C fluorescence or heterogeneity when overall lamin A/C expression increases. The inset shows that the bin span is narrow for low lamin A/C expression and wide for higher lamin A/C levels (*n*=44; data from three independent experiments).

While overall lamin A/C expression levels are typically considered for overall nuclear compliance, we find that the spatial distribution of lamin A/C is not uniform in the nuclear envelope (Fig. 3A,B). This inhomogeneity also increases with overall lamin A/C expression, as revealed by plotting the variance of local lamin A/C fluorescence intensity as a function of overall expression (Fig. 3C; Fig. S3). The histogram of local high lamin A/C fluorescence obtained from high-resolution images shows a narrow and high peak at lower lamin A/C fluorescence levels but the peak broadens and flattens with increase in local lamin A/C fluorescence levels, indicating that lamin A/C distribution gets wider or heterogenous with increase in lamin A/C expression (Fig. 3D). The slope calculated from Fig. 3D was 0.02, which indicates that with unit increase in lamin A/C fluorescence, the heterogeneity increases by 2%. The inset in Fig. 3D shows the bin span to be narrow for low lamin A/C expression and wide for cells expressing higher levels of lamin A/C.

**Fig. 4.**
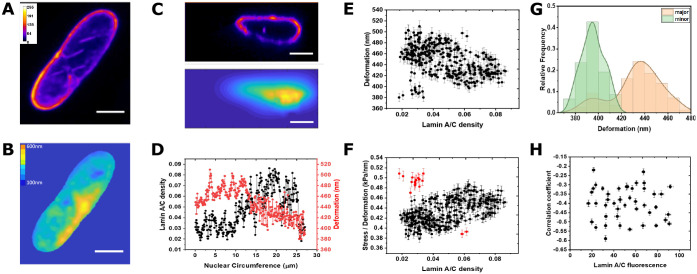
**Local nuclear deformation as a function of lamin A/C distribution in 3T3 fibroblasts.** (A) Lamin A/C distribution along the nuclear membrane in the *x-y* plane. (B) Example strain *x-y* plane map of the 3T3 nucleus shown in A under 193.3 kPa stress. (C) Lamin A/C distribution (top) and *y-z* strain map (bottom) along the nuclear membrane. Scale bars: 5 µm. (D) Ratio of local lamin A/C fluorescence at the membrane to total nuclear fluorescence (lamin A/C density) and nuclear deformation along the nuclear membrane. (E) Deformation plotted against the ratio of local lamin A/C fluorescence at the membrane and total nuclear fluorescence. (F) Ratio of stress to deformation (effective stiffness) plotted against the ratio of local lamin A/C fluorescence at the membrane and total nuclear fluorescence. Results in E and F are for three measurement replicates on the one example experiment, as mean±s.e.m. (G) Statistical distribution of overlaid major and minor axis. (H) The correlation coefficient between local deformation and local lamin A/C (ignoring outliers marked in red) fluorescence was negative showing anticorrelation but with no relationship with total lamin A/C expression. In H, *n*=45; data from three independent experiments and are mean±s.e.m.

We then characterized the relationship between local lamin A/C expression and local nuclear deformation by quantifying the lamin A/C distribution via fluorescence and the nuclear deformation map in the *x-y* and *z*-plane along the nuclear membrane using FIDVC (see Materials and Methods) (Fig. 4A–C). These images suggest an inverse relationship between spatial variations of lamin A/C and deformation. To quantify the spatial variations, we plotted lamin A/C fluorescence with respective deformations along the nuclear circumference, showing the deformation as a function of the fraction of lamin A/C present at the nuclear membrane (Fig. 4D,E). We observed the least nuclear deformation in regions of highest lamin A/C expression and maximum deformation at regions of lowest lamin A/C expression. The ratio of applied osmotic stress to local nuclear deformation as a function of the lamin A/C expression yields the effective contribution of lamin A/C to nuclear stiffness; this reveals that the stiffness increases as lamin A/C expression increases locally (Fig. 4F). While this finding is consistent in all cells measured, some cells also presented low deformations in regions of low lamin A/C expression (see Fig. S4), which may be attributed to the mechanical contributions of the underlying chromatin (Stephens et al., 2017). Chromatin structure may also contribute to the observed mechanical anisotropy of the nucleus along the major and minor axes of the nucleus, where the minor axis is deformed slightly more than the major axis (Fig. 4G). The correlation coefficient between local deformation and local lamin A/C fluorescence was found to be negative for all the cells, clearly showing an anti-correlation between them, but we did not find any relationship between the correlation coefficient values and the total lamin A/C expression (Fig. 4H). This variation in anticorrelation could be due to the effect on the underlying chromatin.

Here, we have quantitatively examined the role of lamin A/C in nuclear mechanics. Using osmotic pressure, we applied precisely controlled stresses and measured global volumetric changes. We find that the overall bulk modulus of the nucleus increases with higher expression levels of lamin A/C. Decomposing nuclear compression, we identify anisotropic deformation of the nucleus showing maximum deformation in *z*-axis followed by the minor axis and then the major axis. Inspecting the distribution of lamin A/C more closely, we found distinct spatial variations in concentration within the nuclear envelope; interestingly, the heterogeneity of lamin A/C density increased with overall expression level. We suspected that these spatial variations in concentration would lead to similar spatial variations in nuclear stiffness. By quantifying the spatial variations of lamin A/C density and comparing this with a submicron resolution deformation map of the nucleus, we indeed found a strong inverse correlation between local submicron lamin A/C concentration and nuclear deformation, demonstrating that the spatial variation of lamin A/C leads to heterogeneous nuclear membrane deformation.

The expression and distribution of lamin A/C are critical in nuclear mechanics, with variations or abnormalities in lamin A/C affecting nuclear stiffness leading to altered nuclear deformation. Pathological conditions such as Hutchinson–Gilford progeria syndrome (HGPS; Goldman et al., 2004) and Emery–Dreifuss muscular dystrophy (EDMD; Håkelien et al., 2008) are related to mutations in the *LMNA* gene, suggesting a potential role of nuclear deformation in nuclear mechanotransduction in the cell, but the relationship between nuclear deformation and how it changes the gene expression is not clear. Evidence for this hypothesis comes from a recent study, where it was shown that when the nucleus is physically compressed, the enzyme histone deacetylase 3 (HDAC3) translocates inside the nucleus. The role of this enzyme is to remove acetyl groups present on histone residues; these acetyl groups neutralize the positive charges on the histones and inhibit their interaction with the negatively charged DNA, resulting in an open conformation of chromatin known as euchromatin (non-compact expressing chromatin conformation). Thus, when the nucleus is mechanically compressed, HDAC3 translocates in the nucleus to remove the acetyl group on the histones, leading to stronger histone–DNA interactions, promoting the non-expressing conformation of chromatin, heterochromatin (Damodaran et al., 2018).

Another factor contributing to nuclear stiffness is the tethering of chromatin itself with the nuclear lamina (Schreiner et al., 2015). Different conformations of chromatin contribute to the mechanical anisotropy of the nuclear envelope (Berendes and Keyl, 1967). While lamin A/C dominates nuclear stiffness under high mechanical stress, under small stresses, chromatin has a crucial role as well (Stephens et al., 2017). Condensed heterochromatin regions are stiffer, and are thus deformed less than euchromatin (Hampoelz and Lecuit, 2011), making chromatin dynamics additionally important in nuclear mechanotransduction. Nevertheless, the physical association between the nuclear lamina and the chromatin, as well as the relationship between nuclear deformation and its direct effect on epigenetic changes, are still unclear and could prove to be essential in relating nuclear mechanics to various pathological conditions.

Previous mechanotransduction studies have shown that mechanical forces can lead to translocation of mechanosensitive proteins such as YAP1 from the cytoplasm to the nucleus, which affects properties like cell proliferation and differentiation in stem cells. Lamin A/C localization could prove to be a critical factor in elucidating this relationship as it directly affects the nuclear stiffness and its deformation. By manipulating lamin A/C expression, relating this to a change in nuclear mechanics, and measuring downstream effects, we may better understand how nuclear stiffness determines cellular fates, thus providing novel mechanics-centered strategies to correct defects in diseases.

## MATERIALS AND METHODS

### Cell culture and modulating nuclear lamin A/C expression

NIH 3T3 cells were obtained from America Type Culture Collection (ATCC) and marked free from any contamination. The cells were cultured following standard protocols in Dulbecco's modified Eagle's medium (DMEM) supplemented with 10% fetal bovine serum (FBS) and 1% penicillin-streptomycin antibiotic. All cells were stably transfected with GFP tagged lamin A/C chromobody (selected with G418 at a concentration of 1000 µg/ml in complete DMEM), which labels the total lamin A/C present on the nuclear membrane without affecting its expression. To overexpress lamin A/C in intact cells, we transfected cells with mCherrytagged plasmid DNA for lamin A/C (Addgene plasmid #55068, deposited by Michael Davison), whereas to suppress lamin A/C expression we transfected the cells with RFP-tagged inducible shRNA construct for lamin A/C (Dharmacon).

### Quantification of lamin A/C expression using antibodies and lamin A/C chromobody

To quantify the total lamin A/C expression in the nucleus, NIH 3T3 fibroblast cells were stably transfected with GFP tagged lamin A/C chromobody (Mayer et al., 2019) (Chromtek). To testify whether the chromobody is an accurate quantitative metric of lamin A/C expression and distribution, we compared its fluorescence with antibody staining (Atto-647N, Sigma) as a gold standard. For this, lamin A/C chromobody-transfected cells were seeded onto gridded coverslips, fixed, permeabilized and stained with Atto-647 lamin A/C antibodies and then imaged with a 63×/1.4 NA oil immersion objective on a confocal microscope (Leica SP8). The stock concentration of the antibody was 1.7 mg/ml with a working concentration of 10 µg/ml. Based on comparisons between chromobody fluorescence and antibody labeling fluorescence in 40 cells, we computed a linear fit with Pearson's *r* value of 0.75. While chromobody and antibody fluorescence have a linear relationship over the majority of the observed range, the *y*-intercept of their fit is negative, suggesting that the antibody has greater sensitivity than the chromobody in detecting very low expression levels of lamin A/C. As with any transfection, the level of GFP-tagged lamin A/C chromobody will differ between cells because transfection efficiency essentially depends on factors like cell density, transfection time, phase of cell cycle, batch of plasmid DNA and DNA concentration. However, to minimize the variation in transfection level, the above-mentioned factors were kept the same in all instances of transfection. Another factor to be taken into consideration while working with lamin A/C antibodies is epitope masking in *xz* plane, as has been shown previously (Ihalainen et al., 2015). However, the present study focuses on quantification performed in the *xy* plane, and thus epitope masking has not been considered as a factor contributing to variation in fluorescence. Fluorescence colocalization was determined using Pearson's correlation coefficient of 0.88±0.04 (mean±s.e.m.) for 10 cells (see Fig. S1B–E).

### Quantification of nuclear bulk moduli

The cells were synchronized in 0.1% FBS-containing medium for 18 h to eliminate the contribution of the cell cycle on lamin A/C expression and distribution (see Fig. S1A). Cell cycle phase identification was undertaken using previously published method by Gomes et al. (2018) (see Fig. S1A). The same set of cells were then attached on coverslips and gradually exposed to medium with 1%, 2.5%, 5%, 7.5% and 10% (w/w) 400 Da polyethylene glycol (PEG 400, Sigma) thus, exerting 847.7 kPa, 936.5 kPa, 1099.2 kPa, 1290.3 kPa and 1514 kPa osmotic stresses one after the other, respectively, for 25 min to reach an equilibrium of compression (Zhou et al., 2009), followed by acquisition of confocal microscopy *xyz*-stacks (63×1.4NA, Leica SP8). The cells compress due to the osmotic pressure exerted by the hypertonic solution causing an efflux of water (Guo et al., 2017), and this change in volume was measured and used to determine the bulk modulus by the relation *B*=−Δ*P*/(Δ*V*/*V*_0_) where, *B* is bulk modulus, Δ*P* is osmotic pressure, Δ*V* change in volume and *V*_0_ is original volume (Khavari and Ehrlicher, 2019).

### 3D volume measurement of nuclei

*xyz* stacks of GFP-tagged lamin A/C chromobody cells were acquired using a 63×/1.4 NA oil immersion objective on a Leica SP8 confocal microscope with a *z*-step size of 0.3 µm. 3D visualization and measurement were carried out using ImageJ software. Before 3D measurement, the nuclear images were deconvolved using the ‘Iterative deconvolve 3D’ plugin in ImageJ. We measured nuclear volumes by counting the number of voxels of the thresholded nuclei and then multiplied it by the size of each voxel to get the volume of the thresholded region. To measure the major, minor and *z*-axis deformations, we acquired *xyz* stacks for different time intervals post-PEG addition. The stacks were summed in the *z*-dimension using ‘z-project’ plugin in ImageJ, followed by fitting the *z*-projections into an ellipse and calculating the major and minor axis for different time intervals.

### Nuclear strain mapping

To measure the local strain on the nuclear membrane, we captured images of the GFP-tagged lamin A/C chromobody nucleus pre- and post-exposure to 2.5% PEG 400 (936.5 kPa). To quantify the local deformation, a custom-made MATLAB code (Bar-Kochba et al., 2015) was used. Briefly, the code utilizes a fast Fourier transform (FFT) based cross-correlation formulation in conjunction with the iterative deformation method (IDM). FIDVC calculates the displacement between the images of GFP tagged nucleus by tracking the pixel intensities of chromobody fluorescence. We mapped these local displacements with the lamin A/C expressions in 3D space to determine the relationship between lamin A/C spatial distribution and nuclear deformation. For the purpose of calculating the correlation coefficient between lamin A/C density and local nuclear deformation, the outliers have been ignored. The outliers were identified by plotting the residual plot using OriginPro software. Based on the residual values, the outliers were identified as points with a standardized residual value higher than +2 or less than −2.

## Supplementary Material

Supplementary information

Reviewer comments
