# Supplementary material for: Spatial distribution of lamin A/C determines nuclear stiffness and stress-mediated deformation
Source: J Cell Sci. 2021 May 24;134(10):jcs248559. doi: 10.1242/jcs.248559 (PMC8186481; doi:10.1242/jcs.248559)
Supplement: Supplementary information [file joces-134-248559-s1.pdf]

Supplementary information

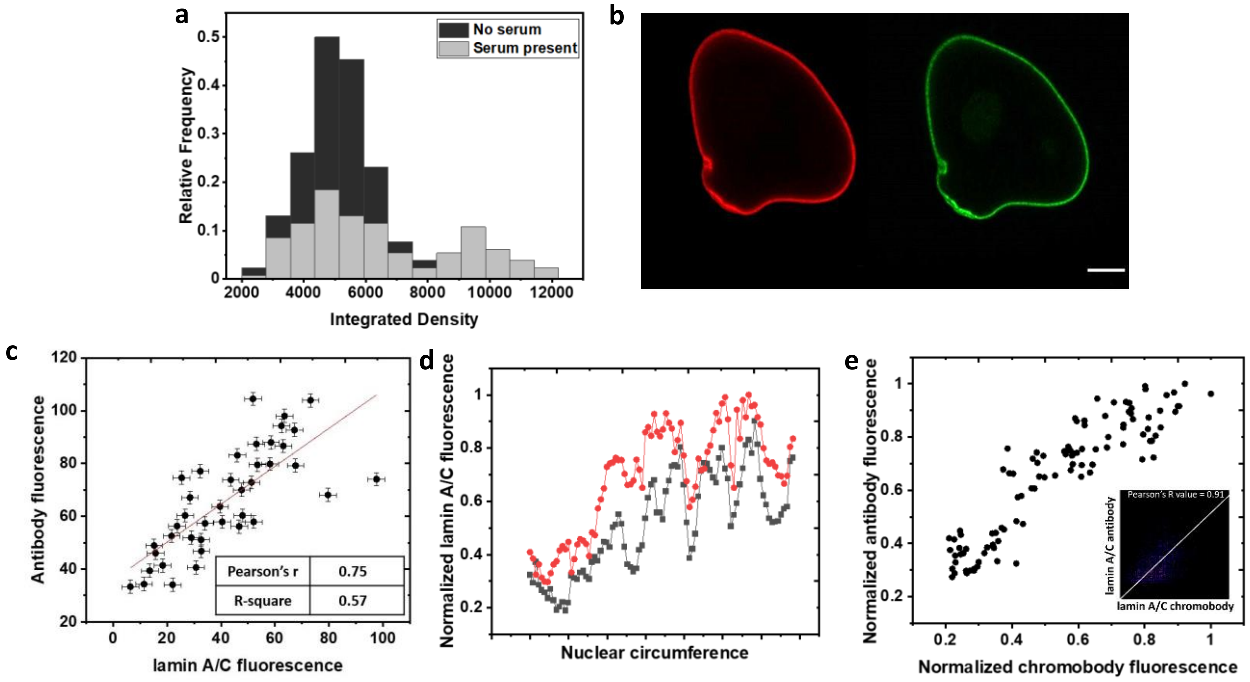

Figure S1. **Spatial correlation between lamin A/C antibody and lamin A/C chromobody in synchronized cells.**

a, Narrow peak of fluorescence in serum starved cells indicate that cells are in the same cell cycle phase compared to a broader peak in cells grown in serum containing media to reduce the variation in overall lamin A/C expression due to different phases of cell cycle (n=130; data from three independent experiments). **b,** Nucleus stained with antibody tagged with Atto-647N (left) and GFP-tagged lamin A/C chromobody (right) (scalebar = 5µm). **c,** Relationship between overall antibody and chromobody fluorescence across unique cells (n=40; data from two independent experiments) showing strong correlation in overall antibody and chromobody fluorescence. **d,** lamin A/C antibody (black) and lamin A/C chromobody (red) fluorescence of the same points along the nuclear circumference indicating local lamin A/C fluorescence to be correlated. **e,** Correlation between antibody and chromobody fluorescence of the same points along the nuclear circumference from **S1b** and an example of colocalization analysis of lamin A/C chromobody and lamin A/C antibody has been shown in the inset indicating a strong correlation even between local antibody and chromobody fluorescence.

Journal of Cell Science • Supplementary information

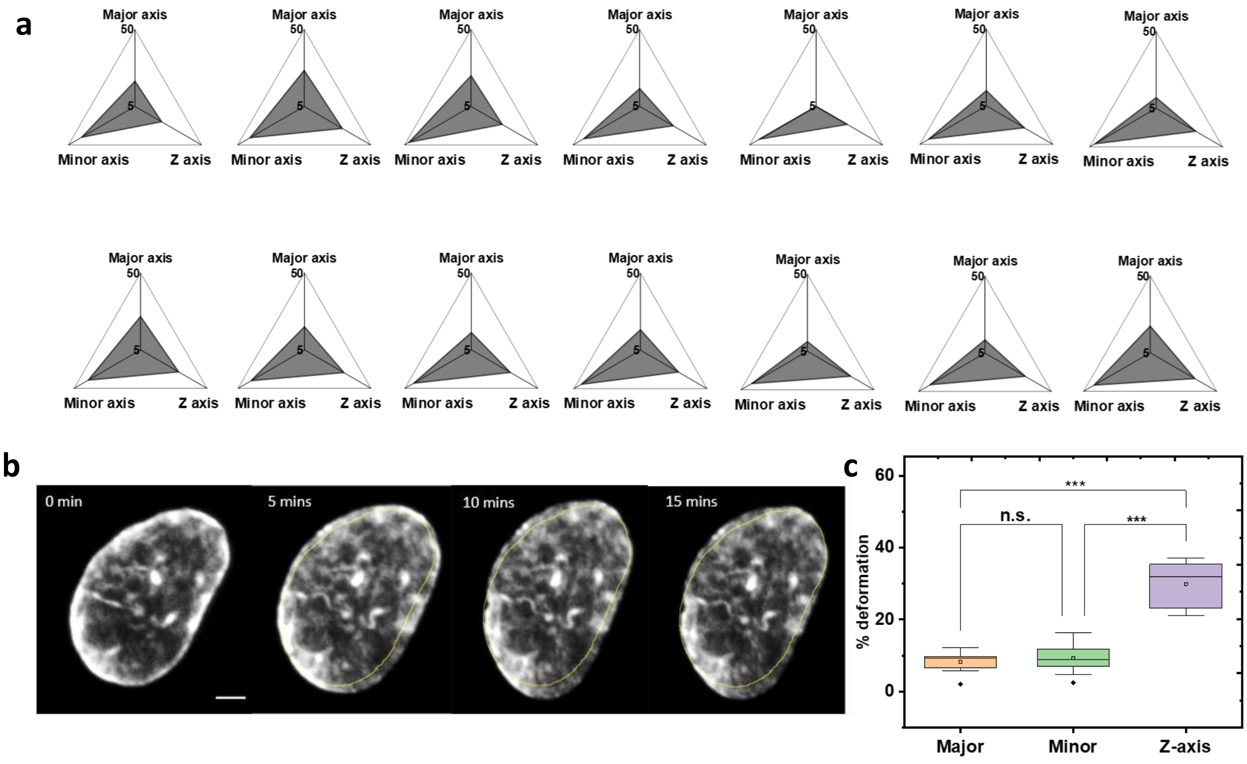

Figure S2. Anisotropy in nuclear compression. a, Nuclear deformation of 14 nuclei (data from a single experiment) along major, minor and z-axis showing the largest deformation along the z-axis. **b,** Z-projections of wild type nucleus stained with lamin A/C chromobody at different time intervals with the original zero pressure image overlaid as a visual aid (scalebar = 2µm). **c**, percent nuclear deformation in nuclei that have been removed from cells and isolated, indicating more pronounced deformation in Z axis (n =10 cells, ***p<=0.001; n.s.= non-significant; data from a single experiment)

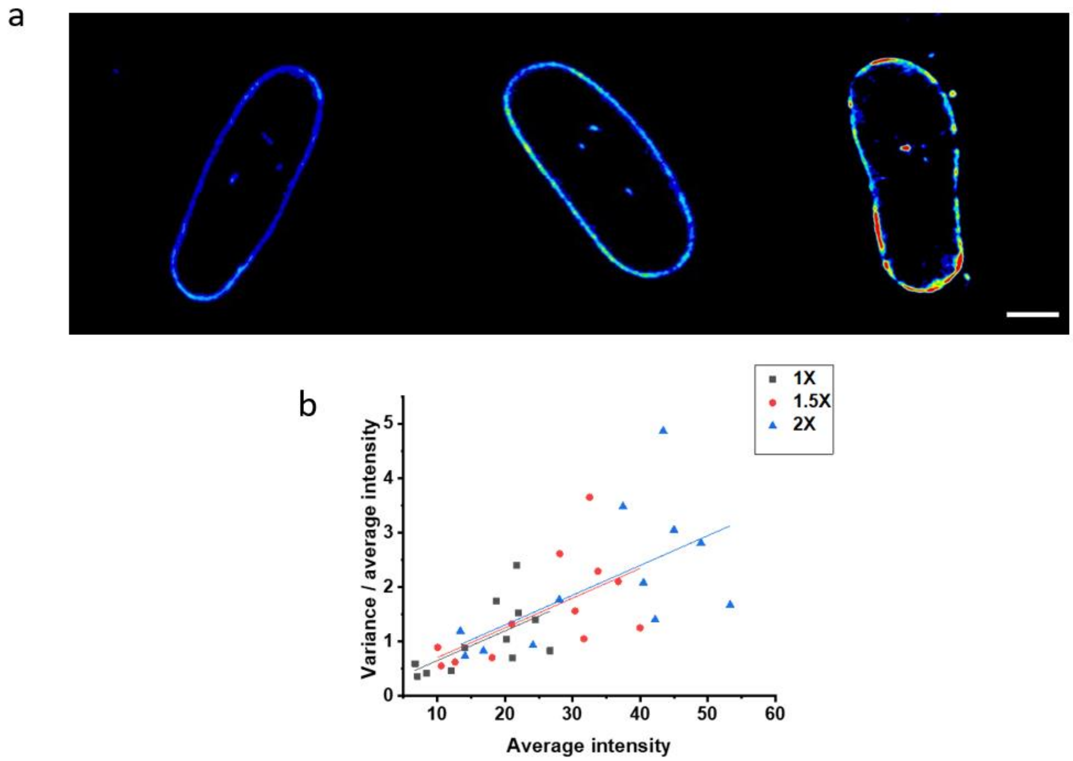

Figure S3. Local nuclear deformation as a function of lamin A/C protein in 3T3 fibroblast cells. a, Increasing lamin A/C heterogeneity with an increase in lamin A/C fluorescence (scale bar = 5μm)**. b,** Normalized variance of lamin A/C chromobody fluorescence (n=12; data from a single experiment) showing that spatial heterogeneity of lamin A/C increases with expression levels and the slope does not change after increasing imaging gain by factors of 1.5 and 2, illustrating that imaging parameters do not increase lamin A/C heterogeneity quantification.

Journal of Cell Science • Supplementary information

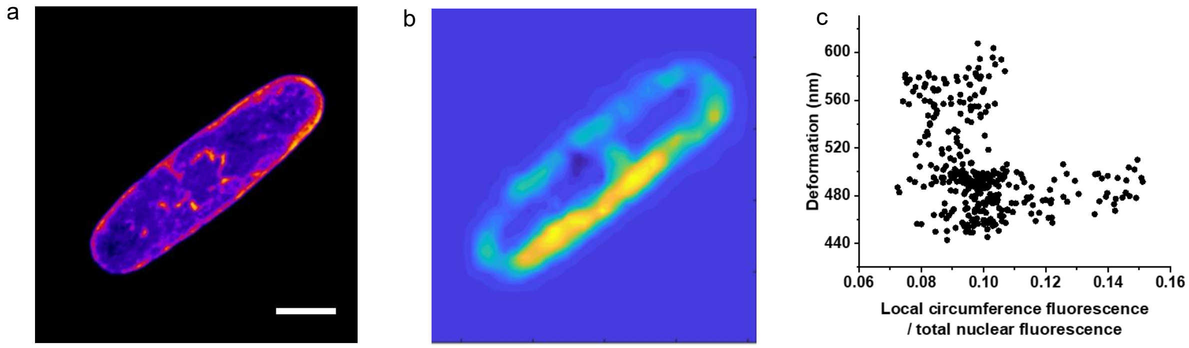

Figure S4. Example local nuclear deformation as a function of lamin A/C. a, lamin A/C distribution along the nuclear circumference in the X-Y plane (scale bar = 5μm). **b,** Nucleus strain map in the X-Y plane. **c,** Nuclear deformation as a function of lamin A/C fluorescence showing a third population with less local deformation in areas of high lamin A/C density

Journal of Cell Science • Supplementary information
