# Supplementary material for: Spatial distribution of lamin A/C determines nuclear stiffness and stress-mediated deformation
Source: J Cell Sci. 2021 May 24;134(10):jcs248559. doi: 10.1242/jcs.248559 (PMC8186481; doi:10.1242/jcs.248559)
Supplement: Reviewer comments [file joces-134-248559_review_history.pdf]

Spatial distribution of lamin A/C determines nuclear stiffness and stress-mediated deformation

Luv Kishore Srivastava, Zhaoping Ju, Ajinkya Ghagre and Allen J. Ehrlicher
DOI: 10.1242/jcs.248559

Editor: Andrew Ewald

Review timeline

Original submission:	12 May 2020
Editorial decision:	11 Jun 2020
Rebuttal received:	30 Jun 2020
Rebuttal decision:	1 July 2020
First revision received:	6 November 2020
Editorial decision:	9 December 2020
Second revision received:	19 January 2021
Editorial decision:	26 February 2021
Third revision received:	22 March 2021
Accepted:	14 April 2021

Original submission

First Decision Letter

MS ID#: JOCES/2020/248559
MS TITLE: Spatial distribution of lamin A determines nuclear stiffness and stress-mediated deformation
AUTHORS: Luv Kishore Srivastava, Zhaoping Ju, Ajinkya Ghagre, and Allen J. Ehrlicher
ARTICLE TYPE: Short Report

As you will see from their reports, the three reviewers had very different views of the manuscript. Reviewer 3 thought the manuscript presented some clear and solid results but raised significant concerns about the novelty of the work relative to prior publications, in particular the anisotropy of nuclear stiffness. Reviewer 2 had major concerns about diverse technical aspects of the experiments and the relatively low number of cells analyzed. This reviewer also raised significant issues regarding the extent of advance over prior publications describing the role of lamins in regulating nuclear deformability and also the spatial role of lamins within the cell. There were also multiple questions raised as to whether the intended variables were being fully isolated (e.g. osmotic pressure at the plasma membrane vs. the nucleus). I do not see a path to address these fundamental concerns within a normal revision period.

I am very sorry to give you such disappointing news, but we are currently under great pressure for space and it takes a very enthusiastic recommendation by the referees for a manuscript to be accepted.

I do hope you find the comments of the reviewers helpful in allowing you to revise the manuscript for submission elsewhere, and many thanks for sending your work to Journal of Cell Science.

© 2021. Published by The Company of Biologists under the terms of the Creative Commons Attribution License (https://creativecommons.org/licenses/by/4.0/).

Reviewer 1

Advance summary and potential significance to field

This manuscript by Srivastava et al., studied the relation of nuclear compressibility/stiffness, and Lamin concentration. In recent years, Lamin has been identified as a critical player in nuclear protection, and cell functionality such as stem cell differentiation. Mechanics of the nucleus is considered a very relevant parameter involved in such regulation. Moreover, cell and nuclear volume are known to have crucial impact on cell behavior as well. How cell and nuclear volume react to external loading is critical for cells during a variety of physiological and pathological processes. However, how the bulk mechanics and compressibility of the cell nucleus are related to the lamin expression remains unknown. Therefore, this paper add a great value to the cell mechanics and mechanobiology field. The paper is well written, and results are nicely presented. I recommend this paper to be published. However, I do have some minor comments.

Comments for the author

1) Fonts are quite small in many figures. It's hard to read if not zoom in by a lot.
2) Some figures need error bars, such as Fig 1 b-d, Fig 2c.
3) The paper measures nuclear volume changes and calculated the bulk modulus which is very nice. However, in the abstract, it's mentioned that the bulk modulus is anisotropic. This is a bit confusing. As the bulk modulus is about volume change, it should be isotropic. I understand that the authors use anisotropic bulk modulus to suggest anisotropic bulk deformation under isotropic pressure. However, it's not typically presented this way, and bulk modulus is always used as an isotropic material parameter even for anisotropic materials. This should at least be commented in the discussion to make sure the implication is clear.

Reviewer 2

Advance summary and potential significance to field

The authors have submitted a manuscript in which they use osmotic pressure and a fluorescent chromobody to investigate the role of lamin A/C on nuclear stiffness and deformation. They report a strong correlation between the levels of lamin A/C and the nuclear bulk modulus, and also a correlation between local lamin A/C levels and deformation. While the manuscript contains some potentially interesting findings, particularly the anisotropic nuclear deformation, it has several substantial limitations, including the novelty, methodology, and interpretation of the results.

Comments for the author

First, the role of lamin A/C on nuclear stiffness and deformability is already well recognized, and thus the manuscript provides few new insights. Contrary to the author's statements in the abstract and introduction, several groups have already performed quantitative measurements on the role of lamin A/C on nuclear deformability, example, Lammerding et al. JBC 2006 (Figure 4) and Swift et al. Science 2013 (Figure 7). In addition, the spatial role of lamins has already been investigated in previous publications, such as Zwerger et al. J Cell Sci 2015 and Nmezi et al. PNAS 2019.

The current manuscript is very descriptive, and does not adequately address the underlying molecular mechanisms, or alternative explanations. Given that the experiments rely on nuclear compression, rather than expansion/tension, it is likely that chromatin will play an important role in the mechanical response (see for example Stephens et al. MBoC 2017). Since lamin A/C can affect chromatin organization and tethering of chromatin to the nuclear lamina, it is possible that the observed effects reflect these indirect effects of lamin on chromatin organization. While the authors briefly discuss this, they do not include any experiments to address this question, which would help to interpret their findings.

There are several substantial concerns regarding the technical approach and interpretation of the findings. Notably, the manuscript almost completely lacks any statistical analysis on the significance of the findings, e.g. whether differences or correlation coefficients are statistically

© 2021. Published by The Company of Biologists under the terms of the Creative Commons Attribution License (https://creativecommons.org/licenses/by/4.0/).

significant, etc. Furthermore, most graphs appear to depict results from single cells, and the experiments are limited to a very small number of cells (10 or less), and it is unclear in how many independent experiments these data were generated. Without further information (and likely a much larger number of cells), these results cannot be adequately interpreted.

The authors use hyperosmotic solution with 400 Da PEG to compress nuclei. They list the osmotic pressure for the various PEG concentrations, presumably from calculations, but one has to assume that this is the osmotic pressure at the cell plasma membrane. At the same time, it appears that the authors equate this osmotic pressure to the mechanical stress on the nucleus when they calculate the bulk modules of the nucleus based on the volume change. Since the osmotic pressure on the nucleus is expected to be substantially different from the osmotic pressure at the plasma membrane, and the cytoskeleton likely alters the mechanical stress on the nucleus, it is very difficult to interpret the findings, which would require an estimate of the actual mechanical stress on the nucleus. This may explain why the authors arrive at a bulk modulus for the nucleus (~2-3 MPa, Figure. 1) that is about 100- to 10000-fold higher than most other estimates of nucleus stiffness (~100 Pa – 10 kPa). Furthermore, it is unclear if the observed anisotropic nuclear deformation indeed reflects anisotropic nuclear material properties, or anisotropic stress on the nucleus, due to anisotropic cellular organization and deformation. To answer the latter question, the authors should at a minimum provide information on the cellular deformation in the x-, y- and z-axis and potential correlation with nuclear deformation.

The authors should also clarify in their description of methods whether individual cells were subjected to increasing PEG concentrations, or if different cells were used for different experiments. In other words, do the results shown in individual plots in Fig. 1b, c, and f represent individual cells, or averages from multiple cells, in which case error bars should be included. This is not clear from the current description of the Methodology.

The authors use the Chromotek chromobody to measure levels of lamin A/C in living cells. Since the fluorescence levels of the chromobody will directly correlate with the expression levels of the chromobody and not just the lamin A/C levels, it is difficult to make cell-to-cell comparisons. The fact that the authors use stably modified cell lines, together with the data presented in Suppl. Fig. 1d, alleviate some of these concerns. However, the data in Suppl. Fig. 1d shows that in some cells, the antibody staining and GFP-chromobody intensities can deviate substantially, which raises concerns for data based on a very small number of cells, as is the case in most of the figures. At a minimum, the authors should consider normalizing the GFP-chromobody fluorescence intensity at the nuclear rim to the whole-cell GFP fluorescence intensity to account for variations in chromobody intensity and repeat experiments in a much larger number of cells.

In their interpretation of the local variations in lamin A/C levels along the periphery, the authors should consider that some of the variation may arise from epitope masking or limited chromobody/antibody accessibility, rather than actual differences in lamin levels, as described for example by Ihalainen et al (Nature Materials 2015). The authors should revise their interpretation accordingly.

In Figure 4, the authors plot the local nuclear deformation vs. the local circumference fluorescence of the lamin A/C chromobody. It is not clear how they relate local deformation, which is measure within an area (and may include deformation in the nuclear interior, as seen in Fig. 4b, c), to a fluorescence intensity values along a line at the nuclear periphery. The authors should provide a more detailed description in the Methodology section, but also conceptually explain how they can relate (local) area deformation measurements to line segment fluorescence intensities.

The methods section describes the use of knockdown and overexpression to modulate lamin A/C levels, but the results shown in the figures do not indicate the use of these cells. If the results include data from cells with lamin A/C depletion or mCherry-lamin A overexpression, this should be clearly indicated in the figures.

The authors state that they used serum starvation (0.1% FBS) to arrest the cell cycle. They should provide corresponding cell cycle data (e.g. DNA content assay) to show the actual cell cycle distribution before and after serum starvation.

© 2021. Published by The Company of Biologists under the terms of the Creative Commons Attribution License (https://creativecommons.org/licenses/by/4.0/).

The authors refer to lamin A throughout the entire manuscript. However, the chromobody recognizes both lamins A and C, not just lamin A, and shRNA mediated depletion will affect both lamins A and C. The authors should revise the manuscript accordingly.

Additional minor issues:

Page 2, line 40. The statement implies that lamins are inner nuclear membrane proteins, which is not correct. The authors should revise this statement.

Page 2, line 41. The authors state that the inner nuclear membrane is held in place by nuclear lamina. This is not correct, as lamin deficient cells (including triple lamin knockout cells) have a normal inner nuclear membrane, and not all inner nuclear membrane proteins rely on interactions with the nuclear lamina. The authors should revise the statement accordingly.

<u>Reviewer 3</u>

Advance summary and potential significance to field

Spatial distribution of lamin A determines nuclear stiffness and stress-mediated deformation" by Srivastava et al. investigates the correlation between nuclear stiffness and lamin A expression at a quantitative level. The author tested the dependence of nuclear stiffness on lamin A expression by measuring the nuclear bulk moduli upon osmotic stress generated by 400 Da PEG. Although the influence of lamin A on nuclear stiffness has already been studied extensively, the author provides some new interesting results on the bulk compressibility of the nuclei, particularly the data showing the changes in nuclear volume as function of time for lamin A expression. The author also assessed the anisotropic nature of nuclear deformation, as well as the inhomogeneous spatial distribution of lamin A in the nuclear envelope. Overall, the paper provides clear and solid results. More specific comments below:

Comments for the author

1. The anisotropic aspect of nuclear stiffness per se is not a novel result. Some references should be added (see below) probably in the introduction, as well as a statement clarifying the distinctiveness of these results.
- Haase, K., Macadangdang, J., Edrington, C. et al. Extracellular Forces Cause the Nucleus to Deform in a Highly Controlled Anisotropic Manner. Sci Rep 6, 21300 (2016).
- Tremblay D, Andrzejewski L, Leclerc A, Pelling AE. Actin and microtubules play distinct roles in governing the anisotropic deformation of cell nuclei in response to substrate strain. Cytoskeleton (Hoboken). 2013;70(12):837-848).
2. The spatially heterogeneous distribution of lamin A in the nuclear envelope seems a novel result.
3. Figure 4c: scale bar is missing.
Figures 3b and 4d: numbers and units are missing at the nuclear circumference axis.
Lines 138-140: "A linear relationship could be seen when we plotted bulk moduli as a function of lamin A fluorescence clearly a direct relationship between lamin A expression and nuclear rigidity": a comma is needed before "clearly"; it would improve the redeability of the sentence.

<u>Rebuttal Letter</u>

Dear Dr Ewald,

Thank you for your email and time in reviewing our manuscript, as well as that of the reviewers; I understand that your decision is to reject the manuscript based on reviewer 2’s critique, and it is abundantly clear that this reviewer is an expert in lamin biology.

© 2021. Published by The Company of Biologists under the terms of the Creative Commons Attribution License (https://creativecommons.org/licenses/by/4.0/).

Before finalizing this decision, would you please allow us to explain why aside from sample size and statistical analysis comments which we were able to address, reviewer 2's comments are in fact factual errors based on subtle misinterpretations of previous work and ours?

In the attached rebuttal we have carefully considered and addressed all of reviewer 2's comments point by point, and find no conceptual conflict; if you agree that our rebuttal has addressed these critiques, would you please allow us two months to submit a revised manuscript that provides the larger sample size requested by the reviewer for their further evaluation, as we were only able to start resuming lab work last week?

Thank you for your consideration and best regards,

Allen Ehrlicher

Rebuttal Response

MS ID#: JOCES/2020/248559

MS TITLE: Spatial distribution of lamin A determines nuclear stiffness and stress-mediated deformation

AUTHORS: Luv Kishore Srivastava, Zhaoping Ju, Ajinkya Ghagre, and Allen J. Ehrlicher
ARTICLE TYPE: Short Report

Thank you for your recent letter. I understand how disappointing my initial decision must have been. I appreciate your noting that the comments of Reviewer 2 made my decision seem reasonable and your careful outlining of your counter-arguments to each point. I can see your perspective on each point and it does appear that overbroad claims were made with regards to what has been previously published in this area. I have reversed my decision and will allow revision. Since your arguments may, or may not, convince reviewer 2, I will add another reviewer in revision who is specifically an expert in mechanics of the nucleus. I have one that I work with regularly who can provide a fresh perspective for me that may counterbalance (or could reinforce) the perspective of current reviewer 2. Since that will be months from now, please send me an email when you resubmit reminding me to do so. Please also feel free to reach out if you need an extended revision timeline.

First revision

Author response to reviewers' comments

Reviewer (black)
Authors (blue)
New/modified data
(red)

Reviewer 1 Advance Summary and Potential Significance to Field:
This manuscript by Srivastava et al., studied the relation of nuclear compressibility/stiffness, and Lamin concentration. In recent years, Lamin has been identified as a critical player in nuclear protection, and cell functionality such as stem cell differentiation. Mechanics of the nucleus is considered a very relevant parameter involved in such regulation. Moreover, cell and nuclear volume are known to have crucial impact on cell behavior as well. How cell and nuclear volume react to external loading is critical for cells during a variety of physiological and pathological processes. However, how the bulk mechanics and compressibility of the cell nucleus are related to the lamin expression remains unknown. Therefore, this paper adds a great value to the cell mechanics and mechanobiology field. The paper is well written, and results are nicely presented. I

© 2021. Published by The Company of Biologists under the terms of the Creative Commons Attribution License (https://creativecommons.org/licenses/by/4.0/).

recommend this paper to be published. However, I do have some minor comments.

Reviewer 1 Comments for the Author:
Fonts are quite small in many figures. It's hard to read if not zoom in by a lot.

We thank the reviewer for the suggestion. The font size has been increased to improve legibility.
Some figures need error bars, such as Fig 1 b-d, Fig 2c.
We have included error bars in plots Fig 1 b-d and Fig 2c and also in other plots missing error bars.

The paper measures nuclear volume changes and calculated the bulk modulus which is very nice. However, in the abstract, it's mentioned that the bulk modulus is anisotropic. This is a bit confusing. As the bulk modulus is about volume change, it should be isotropic. I understand that the authors use anisotropic bulk modulus to suggest anisotropic bulk deformation under isotropic pressure. However, it's not typically presented this way, and bulk modulus is always used as an isotropic material parameter even for anisotropic materials. This should at least be commented in the discussion to make sure the implication is clear.

We thank the reviewer for pointing out this potential confusion. Indeed, the bulk modulus is normally associated with isotropic deformation, and is the basis for which we calculate the quantitative bulk moduli, however, in our investigation we found that the deformation was anisotropic. We have revised this commentary to describe that the “deformation” rather than “modulus” is anisotropic, as that is more accurate physically and hopefully more clear conceptually (line13 and page 1).

Reviewer 2 Advance Summary and Potential Significance to Field:
The authors have submitted a manuscript in which they use osmotic pressure and a fluorescent chromobody to investigate the role of lamin A/C on nuclear stiffness and deformation. They report a strong correlation between the levels of lamin A/C and the nuclear bulk modulus, and also a correlation between local lamin A/C levels and deformation. While the manuscript contains some potentially interesting findings, particularly the anisotropic nuclear deformation, it has several substantial limitations, including the novelty, methodology, and interpretation of the results.

Reviewer 2 Comments for the Author:
First, the role of lamin A/C on nuclear stiffness and deformability is already well recognized, and thus the manuscript provides few new insights.

While it has been broadly reported that lamin A/C influence nuclear stiffness, we were unable to find any report of the **quantitative relationship between lamin A/C expression or distribution and nuclear stiffness**. As we describe below, none of the references cited by the reviewer address this relationship.

Contrary to the author’s statements in the abstract and introduction, several groups have already performed quantitative measurements on the role of lamin A/C on nuclear deformability, example, Lammerding et al. JBC 2006 (Figure 4) and Swift et al. Science 2013 (Figure 7)

We thank the reviewer for pointing out these articles, however, **none of these quantify overall nuclear stiffness or moduli as a function of Lamin A/C expression, nor the spatial variance of deformability, modulus, or stiffness based on Lamin A/C density**.
For clarity, the articles cited by the reviewer are reproduced below:

[NOTE: We have removed a figure which was provided for the referees. It showed Fig. 4 from **Lammerding et al.** (2006) Lamins A and C but not lamin B1 regulate nuclear mechanics. *Journal of Biological Chemistry* **281**, 25768-25780. (DOI: 10.1074/jbc.M513511200)]

Fig 4 of Lammerding et al, no measurement of stiffness or modulus is given on any axis. In Panel E, strain is measured as function of relative lamin A, B1, B2, and C expression levels, but strain is not a stiffness characterization. Additionally the spread in the data in panel E make any mechanical relationship difficult to infer.

© 2021. Published by The Company of Biologists under the terms of the Creative Commons Attribution License (https://creativecommons.org/licenses/by/4.0/).

Fig 7 of Swift et al.

Fig 7 of Swift et al. (below) reports the Shear Modulus and viscosity of the nucleus as a function of Lamin A: B ratio, **not as a function of Lamin A/C expression or density**

[NOTE: We have removed a figure which was provided for the referees. It showed Fig. 7 from **Swift et al.** (2013) Nuclear lamin-A scales with tissue stiffness and enhances matrix-directed differentiation. *Science*, **341(6149).** (DOI: 10.1126/science.1240104)]

In addition, the spatial role of lamins has already been investigated in previous publications, such as Zwerger et al. J Cell Sci 2015 and Nmezi et al. PNAS 2019.

We are unclear about what **spatial role** the reviewer is suggesting. Zwerger et al (https://pubmed.ncbi.nlm.nih.gov/26275827/) demonstrate that disruptions to lamin proteins result in misshapen nuclei, but offers **no description of the impact of spatial lamin variation on mechanical properties**.
The only partially related mechanical measurement is the observation that normalized nuclear strain varies with overall lamin isoform (Fig 5C from the Zwerger manuscript, [NOTE: We have removed a figure which was provided for the referees. It showed Fig. 5C from Zwerger et al. (2015) Altering lamina assembly reveals lamina-dependent and-independent functions for A-type lamins. Journal of Cell Science, 128(19), 3607-3620. (DOI: 10.1242/jcs.171843)]), yet this does not relate the spatial distribution of lamins with local mechanical properties. This characterization is completely unrelated to **our measurement that relates to the sub-micron density of lamin A with local nuclear stiffness.**

The current manuscript is very descriptive and does not adequately address the underlying molecular mechanisms, or alternative explanations.

We thank the reviewer for noting that our manuscript is descriptive, but are unclear about what underlying mechanisms are sought or missing. As the reviewer pointed out, lamins are recognized as structural proteins that contribute to nuclear stiffness but studies to date have focused on comparing gross nuclear stiffness changes in ensemble population lamin A/C expression but have not examined the quantitative dependence of nuclear stiffness on lamin A/C expression, or the impact of the spatial distribution of lamin A/C of deformation. Here we quantify how the overall expression and spatial density of those structural proteins impact global and local nuclear stiffness, resulting in heterogeneous deformation of the nucleus under isotropic stress.

Given that the experiments rely on nuclear compression, rather than expansion/tension, it is likely that chromatin will play an important role in the mechanical response (see for example Stephens et al. MBoC 2017).

Indeed we agree with the reviewer that chromatin does play a role in the nuclear compressibility, as do other macromolecular structures within the nucleus. Our manuscript does not suggest that lamins **alone** determine nuclear stiffness but reveals their relative contribution to bulk mechanics and local stiffness as mentioned on Page.13, line 261 in the **'discussion'** section of the manuscript.

Since lamin A/C can affect chromatin organization and tethering of chromatin to the nuclear lamina, it is possible that the observed effects reflect these indirect effects of lamin on chromatin organization. While the authors briefly discuss this, they do not include any experiments to address this question, which would help to interpret their findings.

We thank the reviewer for raising this point. Indeed, as pointed out in Stephens et al, the chromatin condensation state does directly influence the mechanics of the nucleus as we concur above, however, this is different from tethering to lamin. We suggest that compression measurements are actually preferable to tension for the very reason of potential lamin-chromatin tethering: Flexible polymers such as lamins have large tensile moduli but buckle and bend readily under compression. Tethering would significantly impact Young's moduli as evaluated through stretching of the nucleus, as not only lamin must be deformed, but the

© 2021. Published by The Company of Biologists under the terms of the Creative Commons Attribution License (https://creativecommons.org/licenses/by/4.0/).

tethered chromatin underneath must be stretched as well- if the chromatin is not tethered, this would result in an easier extension and softer nucleus. In contrast, in compression the chromatin is compressed whether or not it is tethered to the lamin structure, and thus has little potential impact on compression bulk modulus measurements. Nevertheless, we describe this as a potential interaction in the manuscript which may still impact nuclear mechanics and will be an essential subject of study for our next manuscript in which we examine chromatin contributions to nuclear mechanics.

There are several substantial concerns regarding the technical approach and interpretation of the findings. Notably, the manuscript almost completely lacks any statistical analysis on the significance of the findings, e.g. whether differences or correlation coefficients are statistically significant, etc. Furthermore, most graphs appear to depict results from single cells, and the experiments are limited to a very small number of cells (10 or less), and it is unclear how many independent experiments these data were generated. Without further information (and likely a much larger number of cells), these results cannot be adequately interpreted.

We apologize for giving the impression that our findings are not statistically robust; in the manuscript presentation, we did focus on single cell experiments for clarity of methodology and concept. We have included the requested statistical analysis of the studies below.

In the figure to the left (Fig 1d from manuscript) we report a Pearson's coefficient of 0.96 and an R^2 value 0.92 for the correlation between lamin A/C fluorescence (as measured by lamin A/C Chromobody) and bulk modulus, demonstrating the strong quantitative scaling of bulk modulus with lamin A expression level. This high correlation also demonstrates that lamin A dominates the bulk modulus. To our knowledge, such data has never before been reported. These data are based on 8 cells in a single experiment. [NOTE: We have removed a figure which was provided for the referees in confidence.]

The image on the left is Fig.1e in the modified manuscript with a larger sample size of 46 cells with Pearson's coefficient of 0.91 and R^2 value 0.85 between lamin A/C fluorescence and the bulk modulus. With a larger sample size, it remains clear that the bulk modulus increases with increase in the overall lamin A/C fluorescence suggesting a strong relationship between lamin A/C expression and nuclear compliance. [NOTE: We have removed a figure which was provided for the referees in confidence.]

In the figure to the right (Fig 3c from manuscript) we report a Pearson's coefficient of 0.61 and an R^2 value 0.37 for the correlation between normalized lamin A/C fluorescence variance (as measured by lamin A/C Chromobody) and overall average fluorescence of lamin A in the nucleus, demonstrating that the spatial heterogeneity of lamin A increases with overall expression level. The spatial variation of lamin A, however, is likely not solely dependent on lamin A expression, and as such the correlation is not very high. To our knowledge, such data has never before been reported. These data are based on 12 cells in single experiment. [NOTE: We have removed a figure which was provided for the referees in confidence.]

The figure to the right is the new Fig. 3C in the manuscript with a Pearson's correlation coefficient of 0.59 demonstrating that the spatial heterogeneity of lamin A/C increases with overall lamin A/C expression level. [NOTE: We have removed a figure which was provided for the referees in confidence.]

In the figure to the left (Fig 4e from manuscript) correlating the local nuclear membrane deformation with normalized local membrane lamin A fluorescence we report a Pearson's coefficient of -0.38, demonstrating an inverse relationship between deformability and local density of lamin A/C (as measured by lamin A/C Chromobody). This means that denser lamin A/C areas are stiffer. **To our knowledge, such data has never before been reported**. [NOTE: We have removed a figure which was provided for the referees in confidence.]

We find an R^2 value of 0.16 for this correlation, demonstrating wide variance in values, largely driven by the visible outliers in the lower right of the plot. These outliers, where deformation is low, but lamin A density is also low, arise from endpoints of the nucleus along the major axis, and

© 2021. Published by The Company of Biologists under the terms of the Creative Commons Attribution License (https://creativecommons.org/licenses/by/4.0/).

reflect the XYZ anisotropy of nuclear deformation independent of lamin A/C.
Removing these outliers increases the Pearson's coefficient and R^2 to -0.59 and 0.33 respectively. To our knowledge, such data has never before been reported. These data points are for a single cell. The average Pearson's correlation for 10 cells is -0.23 ± 0.08.

The figure on the left is the new Fig. 3h in the revised manuscript showing the average correlation coefficient to be -0.39 ± 0.09. The negative value of correlation is indicative of the anti correlation that exists between lamin A/C density and local deformation. This expands the concept from the single cell (Fig 4e) to that of a statistical population. [NOTE: We have removed a figure which was provided for the referees in confidence.]

In the figure to the left (Fig 2C from manuscript) we apply the one way ANOVA test for statistical significance of nuclear deformation in intact cells, revealing that percent deformations along the major and minor axis within the XY plane are not significantly different ($p>0.05$) whereas the differences between the major axis and Z ($p<=0.001$), and minor axis and Z ($p<=0.001$) are both statistically significant. (n = 9 cells) [NOTE: We have removed a figure which was provided for the referees in confidence.]

The figure on the left is the modification of Fig. 2C in the original manuscript with increased sample size **(n=44 cells)** and statistical analysis. We apply the one way ANOVA test for statistical significance of nuclear deformation in intact cells, revealing that percent deformations along the major and minor axis within the XY plane are not significantly different ($p>0.05$) whereas the differences between the major axis and Z ($p<=0.001$), and minor axis and Z ($p<=0.001$) are both statistically significant. [NOTE: We have removed a figure which was provided for the referees in confidence.]

The authors use a hyperosmotic solution with 400 Da PEG to compress nuclei. They list the osmotic pressure for the various PEG concentrations, presumably from calculations, but one has to assume that this is the osmotic pressure at the cell plasma membrane. It appears that the authors equate this osmotic pressure to the mechanical stress on the nucleus when they calculate the bulk modulus of the nucleus based on the volume change. Since the osmotic pressure on the nucleus is expected to be substantially different from the osmotic pressure at the plasma membrane, and the cytoskeleton likely alters the mechanical stress on the nucleus, it is very difficult to interpret the findings, which would require an estimate of the actual mechanical stress on the nucleus.

In the current manuscript we report calculated values, however, all osmotic stresses have been both calculated and directly measured by our group, and are in good agreement (please see **Khavari & Ehrlicher**, PLoSONE 2019, and the table below taken from that article [NOTE: We have removed a table which was provided for the referees. It showed Table S1 from Khavari & Ehrlicher, (2019) Nuclei deformation reveals pressure distributions in 3D cell clusters. *PLoS One*, **14(9),** e0221753. (DOI: 10.1371/journal.pone.0221753)])

The osmotic pressure at the plasma membrane and nuclear membrane are the same for the following reasons- the cell and the nucleus are porous, permeable membrane-enclosed structures which allows movement of water, ions and small molecules through pore complexes that control the large molecules transfer and act as a molecular sieve. When a porous gel such as the cell or nucleus is exposed to an external solution of macromolecules higher than that in the gel, the difference in concentration is quantified by the partition coefficient (Albro et al, Journal of Biomechanical Engineering 2007), which leads to an osmotic gradient that draws water out of the gel. This leads to a higher pressure on the gel when the concentration of the macromolecule increases in the solution.
Osmotically induced volume changes of the cytoplasm lead to higher intracellular macromolecule concentration within the cytoplasm, which transfers this osmotic stress and compress the nucleus. This means the number of small molecules coming out of the cytoplasm would be equal to the number of water molecules coming out of the nucleus to keep the system in equilibrium. Therefore, when we apply hyperosmotic solutions to the cell, the same forces applied to the cell are transferred to the nucleus.

It is also shown that nuclear compression under hyper osmotic conditions is proportional to intracellular macromolecule concentration, increased molecular crowding within the nucleus (Finan

© 2021. Published by The Company of Biologists under the terms of the Creative Commons Attribution License (https://creativecommons.org/licenses/by/4.0/).

et al, Annals of Biomedical Engineering 2009) Thus, externally applied pressure is transmitted to the nuclei as the cytoplasm is compressed to an equilibrium pressure, as discussed in our previous article where osmotic stress was transmitted not only through the cytoplasm of a single cell but through large multicellular aggregates (Khavari & Ehrlicher, PLoSONE 2019).

The figure on the left (Guo et al PNAS 2017) shows a linear trend between change in **cell and nuclear volume** under hyperosmotic pressure indicating the osmotic pressure is transmitted on to the nucleus in the same proportion. [NOTE: We have removed a figure which was provided for the referees. It showed Figure 6A from **Guo et al.** (2017) Cell volume change through water efflux impacts cell stiffness and stem cell fate. *Proceedings of the National Academy of Sciences*, **114(41),** E8618-8627. (DOI: 10.1073/pnas.1705179114)]

Regarding the role of the cytoskeleton in nuclear moduli, we also measured the moduli of isolated nuclei (included here and as a supplemental figure) to remove the effect of cytoskeleton on the nucleus. Following the procedure detailed in (Guilack et al 2000, Watson et al 2010) this is done by lysing the cells using 0.1% igepal in PBS (a mild detergent). The igepal-PBS solution was supplemented with ATP and protease inhibitor cocktail to maintain the integrity of the nuclear membrane. After lysis, the lysate was centrifuged at 10,000 r.p.m for 20 seconds to obtain the isolated nucleus which was then seeded onto coverslips (#1). Then the isolated nuclei were exposed to different hyperosmotic pressure induced by PEG.

The figures to the left (fig 1c and 1d in manuscript) shows nuclear deformation and bulk moduli in intact cells, whereas the new figure to the right depicts the nuclear deformation and bulk moduli **in nuclei that have been removed from cells and isolated** (n =7 nuclei).The way intact and isolated nuclei compress under nuclear deformation was very similar, though the absolute values of Bulk modulus in case of isolated nucleus was slightly less than the intact nuclei, potentially due to cytoskeletal protection in the cell.

This may explain why the authors arrive at a bulk modulus for the nucleus (~2-3 MPa, Figure. 1) that is about 100- to 10000-fold higher than most other estimates of nucleus stiffness (~100 Pa – 10 kPa).

The 1000x fold difference certainly may seem alarming, but it is because **the moduli are different types**. The reason for the difference in moduli is due to the nature of **bulk moduli** versus **shear (cortical) moduli.** In Guo et al PNAS 2017, these are directly compared in Figure 1 (left), resulting in a 1000x fold difference. We apologize for this easy misunderstanding. [NOTE: We have removed a figure which was provided for the referees. It showed Figure 1A from **Guo et al**. (2017) Cell volume change through water efflux impacts cell stiffness and stem cell fate. *Proceedings of the National Academy of Sciences*, **114(41),** E8618-8627. (DOI: 10.1073/pnas.1705179114)]

Furthermore, it is unclear if the observed anisotropic nuclear deformation indeed reflects anisotropic nuclear material properties, or anisotropic stress on the nucleus, due to anisotropic cellular organization and deformation. To answer the latter question, the authors should at a minimum provide information on the cellular deformation in the x-, y- and z-axis and potential correlation with nuclear deformation.

Thank you for this comment; while we agree that external cell anisotropy may confer some additional stress to the nucleus, in isolated nuclei (where the cytoskeleton and all other cell components have been removed) we observed very similar nuclear stiffness anisotropy (see graph below as new supplemental figure), where the cytoskeleton and all other cell components have been removed. This suggests that the anisotropy is within the nucleus itself.

The figure to **the left** (fig 2C in manuscript) shows Percent deformation nuclear compression in nuclei in cells, whereas the new figure to the **right** depicts the percent nuclear deformation **in nuclei that have been removed from cells and isolated** (n =10 cells, ***p<=0.001; n.s.= non-significant). The anisotropy of deformation in intact nuclei inside the cell in very similar to that observed in intact cells, suggesting that the anisotropy is indeed largely determined by the nucleus rather than the cytoskeleton [NOTE: We have removed a figure which was provided for the referees in confidence.]

© 2021. Published by The Company of Biologists under the terms of the Creative Commons Attribution License (https://creativecommons.org/licenses/by/4.0/).

The authors should also clarify in their description of methods whether individual cells were subjected to increasing PEG concentrations, or if different cells were used for different experiments. In other words, do the results shown in individual plots in Fig. 1b, c, and f represent individual cells, or averages from multiple cells, in which case error bars should be included. This is not clear from the current description of the Methodology.

Thank you for this note- the data in Fig 1 represent increased applied pressure to the same cells, and this is noted in our methods.

The authors use the Chromotek chromobody to measure levels of lamin A/C in living cells. Since the fluorescence levels of the chromobody will directly correlate with the expression levels of the chromobody and not just the lamin A/C levels, it is difficult to make cell-to-cell comparisons. The fact that the authors use stably modified cell lines, together with the data presented in Suppl. Fig. 1d, alleviate some of these concerns. However, the data in Suppl. Fig. 1d shows that in some cells, the antibody staining and GFP-chromobody intensities can deviate substantially, which raises concerns for data based on a very small number of cells, as is the case in most of the figures. At a minimum, the authors should consider normalizing the GFP-chromobody fluorescence intensity at the nuclear rim to the whole- cell GFP fluorescence intensity to account for variations in chromobody intensity and repeat experiments in a much larger number of cells.

The correlation between chromobody and antibody **total nuclear fluorescence** is 0.73 for 40 cells (ignoring the outliers) indicating that the chromobody is accurate in making quantitative cell to cell comparisons of lamin expression using the chromobody, with the antibody as a “gold standard”. [NOTE: We have removed a figure which was provided for the referees in confidence.]

The **colocalization** between **local** chromobody and antibody fluorescence along the nuclear envelope has a mean Pearson’s correlation coefficient of 0.88 for 10 high resolution nuclei images comparing both chromobody and antibody at the same time in nuclei, demonstrating that the chromobody accurately reports the spatial density of lamin A/C.

In their interpretation of the local variations in lamin A/C levels along the periphery, the authors should consider that some of the variations may arise from epitope masking or limited chromobody/antibody accessibility, rather than actual differences in lamin levels, as described for example by Ihalainen et al (Nature Materials 2015). The authors should revise their interpretation accordingly.

We thank the author for pointing out this potential confounding variable which impacts any effort to quantify lamins. We note that in the article in question, the authors quantified lamin A fluorescence variation in apical vs basal planes as imaged from the side “YZ” but illustrate homogeneity in “XY” planes, which is the data set reported in our study. [NOTE: We have removed a figure which was provided for the referees. It showed a section of Figure 1A from **Ihalainen et al.** (2015) Differential basal-to-apical accessibility of lamin A/C epitopes in the nuclear lamina regulated by changes in cytoskeletal tension. *Nature materials*, **14(12)**, 1252-1261. (DOI: 10.1038/nmat4389)]

In Figure 4, the authors plot the local nuclear deformation vs. the local circumference fluorescence of the lamin A/C chromobody. It is not clear how they relate local deformation, which is measure within an area (and may include deformation in the nuclear interior, as seen in Fig. 4b, c), to a fluorescence intensity values along a line at the nuclear periphery. The authors should provide a more detailed description in the Methodology section, but also conceptually explain how they can relate (local) area deformation measurements to line segment fluorescence intensities.

The data in figure 4 represents the local lamin A/C fluorescence on the nuclear membrane and the deformation (displacement) of the same positions on just the nuclear membrane.

The methods section describes the use of knockdown and overexpression to modulate lamin A/C levels, but the results shown in the figures do not indicate the use of these cells. If the results include data from cells with lamin A/C depletion or mCherry-lamin A overexpression, this should be clearly indicated in the figures.

© 2021. Published by The Company of Biologists under the terms of the Creative Commons Attribution License (https://creativecommons.org/licenses/by/4.0/).

We thank the reviewer for pointing this out. All cells have been clearly identified as to how their lamin expression was modified as per the reviewer's suggestion.

The authors state that they used serum starvation (0.1% FBS) to arrest the cell cycle. They should provide corresponding cell cycle data (e.g. DNA content assay) to show the actual cell cycle distribution before and after serum starvation.

We thank the reviewer for suggesting this validation experiment;

As suggested by the reviewer, the validation was done according to previously published methodology (Gomes, Cecil J., et al. "Measuring DNA content in live cells by fluorescence microscopy." Cell division 13.1 (2018): 1-10.). The figure on the left shows the Integrated density (ID) of 40 cells stained with DAPI. The narrow peak at lower ID level for serum starved cells shows that most of the cells were synchronized compared to a broader distribution of ID for cells with serum in the growth media. [NOTE: We have removed a figure which was provided for the referees in confidence.]

The authors refer to lamin A throughout the entire manuscript. However, the chromobody recognizes both lamins A and C, not just lamin A, and shRNA mediated depletion will affect both lamins A and C. The authors should revise the manuscript accordingly.

We thank the reviewer for pointing this out. We sincerely regret any confusion on this aspect of shorthand notation, and have revised the manuscript accordingly.

Additional minor issues:

Page 2, line 40. The statement implies that lamins are inner nuclear membrane proteins, which is not correct. The authors should revise this statement.

We thank the reviewer for pointing this out. The suggested change has been revised in the manuscript accordingly.

Page 2, line 41. The authors state that the inner nuclear membrane is held in place by nuclear lamina. This is not correct, as lamin deficient cells (including triple lamin knockout cells) have a normal inner nuclear membrane, and not all inner nuclear membrane proteins rely on interactions with the nuclear lamina. The authors should revise the statement accordingly.

We thank the reviewer for pointing this out. The suggested change has been revised in the manuscript accordingly.

Reviewer 3 Advance Summary and Potential Significance to Field:
Spatial distribution of lamin A determines nuclear stiffness and stress-mediated deformation" by Srivastava et al. investigates the correlation between nuclear stiffness and lamin A expression at a quantitative level. The author tested the dependence of nuclear stiffness on lamin A expression by measuring the nuclear bulk moduli upon osmotic stress generated by 400 Da PEG. Although the influence of lamin A on nuclear stiffness has already been studied extensively, the author provides some new interesting results on the bulk compressibility of the nuclei, particularly the data showing the changes in nuclear volume as function of time for lamin A expression. The author also assessed the anisotropic nature of nuclear deformation, as well as the inhomogeneous spatial distribution of lamin A in the nuclear envelope. Overall, the paper provides clear and solid results. More specific comments below:

Reviewer 3 comments for the author:

The anisotropic aspect of nuclear stiffness per se is not a novel result. Some references should be added (see below) probably in the introduction, as well as a statement clarifying the distinctiveness of these results.

1) Haase, K., Macadangdang, J., Edrington, C. et al. Extracellular Forces Cause the Nucleus to

© 2021. Published by The Company of Biologists under the terms of the Creative Commons Attribution License (https://creativecommons.org/licenses/by/4.0/).

Deform in a Highly Controlled Anisotropic Manner. Sci Rep 6, 21300 (2016).

2) Tremblay D, Andrzejewski L, Leclerc A, Pelling AE. Actin and microtubules play distinct roles in governing the anisotropic deformation of cell nuclei in response to substrate strain. Cytoskeleton (Hoboken). 2013;70(12):837-848).

We thank the reviewer for pointing these key references from the Pelling lab, which have been added. The suggested change of improving the distinctiveness of the results has been revised in the manuscript "previous studies have also indicated the nuclear deformation to be anisotropic(Haase et al., 2016; Tremblay et al., 2013), but it remains unknown how the spatial heterogeneity of lamin A/C affects nuclear deformation" (line 69 page3).

Figure 4c: scale bar is missing. Figures 3b and 4d: numbers and units are missing at the nuclear circumference axis.

We have added a scale bad to figure 4c, and numbers and units for Figs 3b and 4d.

Lines 138-140: "A linear relationship could be seen when we plotted bulk moduli as a function of lamin A fluorescence clearly a direct relationship between lamin A expression and nuclear rigidity": a comma is needed before "clearly"; it would improve the readability of the sentence.

We have added the suggested comma to the sentence.

Second decision letter

MS ID#: JOCES/2020/248559

MS TITLE: Spatial distribution of lamin A/C determines nuclear stiffness and stress-mediated deformation

AUTHORS: Luv Kishore Srivastava, Zhaoping Ju, Ajinkya Ghagre, and Allen J. Ehrlicher
ARTICLE TYPE: Short Report

We have now reached a decision on the above manuscript.

To see the reviewers' reports and a copy of this decision letter, please go to: https://submit-jcs.biologists.org and click on the 'Manuscripts with Decisions' queue in the Author Area. (Corresponding author only has access to reviews.)

As a reminder, after the first submission I rejected this manuscript because I didn't think that the concerns of the reviewers could be addressed in a standard revision period. You successfully appealed on the basis that you felt they could be addressed, though it might take more time, and requested a counter-balance to the strong opinions of Reviewer 2. I sent it for review, with an additional expert in nuclear mechanics as Reviewer 4. That reviewer has also checked all of the other reviews. I think the process validated both of our positions. There is more remaining after this revision than normal and...I think you will be able to substantively respond to their concerns. As you will see, the reviewers find that some of the issues in the first round have been addressed but highlight significant areas that remain.They suggest, however, that a revised version might prove acceptable, if you can address their concerns. If you think that you can deal satisfactorily with the criticisms on revision, I would be pleased to see a revised manuscript. We would then return it to the reviewers.

We are aware that you may be experiencing disruption to the normal running of your lab that makes experimental revisions challenging. If it would be helpful, we encourage you to contact us to discuss your revision in greater detail. Please send us a point-by-point response indicating where

© 2021. Published by The Company of Biologists under the terms of the Creative Commons Attribution License (https://creativecommons.org/licenses/by/4.0/).

you are able to address concerns raised (either experimentally or by changes to the text) and where you will not be able to do so within the normal timeframe of a revision. We will then provide further guidance. Please also note that we are happy to extend revision timeframes as necessary.

Please ensure that you clearly highlight all changes made in the revised manuscript. Please avoid using 'Tracked changes' in Word files as these are lost in PDF conversion.

I should be grateful if you would also provide a point-by-point response detailing how you have dealt with the points raised by the reviewers in the 'Response to Reviewers' box. Please attend to all of the reviewers' comments. If you do not agree with any of their criticisms or suggestions please explain clearly why this is so.

Reviewer 1

Advance summary and potential significance to field

The authors addressed all my questions and the manuscript reads well now.

Comments for the author

no further comments

Reviewer 2

Advance summary and potential significance to field

The authors have submitted a revised manuscript that addresses several of the previous reviewersÂ' concerns. The authors have now included additional data to increase the robustness of their key findings. The central novelty of the manuscript is the single cell based analysis that shows a strong linear correlation between lamin A/C levels and the nuclear bulk modulus, and the correlation of local nuclear deformation and local lamin A/C levels. These results will be of interest to the cell biology community.

Comments for the author

Nonetheless, several concerns remain to be addressed by the authors to sufficiently support their conclusions and to ensure scientific rigor and reproducibility of the work. For most of their experiments, the authors now include data from larger numbers of cells, e.g. the data provided in Figures 1D, 1E, 2C, 3C, 4H, S1E, which is a substantial improvement. However, other conclusions are still based on an extremely small number of cells, such as the results presented in Figs. 1F, 3D, making their interpretation difficult. For example, based on the results presented in Fig. 1G, the authors postulate a different nuclear dynamic mechanical behavior for cells with a lamin A/C fluorescence value above 16.9, but this conclusions is based on the behavior of just two cells below that threshold, which is insufficient to make such a claim. The authors should either provide additional data to support their conclusions, or revise their statements.

Furthermore, important information on the data used for the analysis is missing. The authors do not include the (required) information in the figure legends on how many times the experiments shown were replicated in the laboratory. Such statements are critical to assess how robust the findings are, given the large variability inherent to many cell biology experiments. The authors should ensure that all figure legends contain this information.

The authors continue to state in their abstract and other sections of the text that “the quantitative relationship between lamin A/C and nuclear deformation is unknown”. This statement is not correct, as numerous groups have shown quantitative relationships between lamin expression and nuclear deformation (e.g. nuclear strain, nuclear deformation, or stiffness). In addition to the publications already pointed out in the previous reviewer’s comments, other examples includes work by Schaepe et al. (Biophys J 2009, Fig. 4) and Swift et al. (Science 2013, Fig. 7; note that

© 2021. Published by The Company of Biologists under the terms of the Creative Commons Attribution License (https://creativecommons.org/licenses/by/4.0/).

although the figure plots the lamin A:B ratio in the x-axis, other experiments in the same manuscript showed that lamin B is essentially constant, and therefore the graph essentially represents a plot of the lamin A levels). The authors should revise their text to state that the relationship between the nuclear bulk modulus and lamin A/C levels had not been determined previously, and that they now provide a single cell (and subcellular cell) based analysis. This would provide a more accurate reflection of the novelty provided by this manuscript.

In the images presented in the manuscript and in the corresponding analysis, the authors show lamin A/C (almost) to be located almost exclusively at the nuclear periphery, even though lamins also form stable structures in the nuclear interior (see for example work by Robert Goldman and Roland Foisner). The authors should comment on whether the lack of nucleoplasmic lamin A/C signal in their data is reflective of the specific cell line and maybe the selection of cells in G0/G1 phase, or whether the deconvolution and image processing contributed to the elimination of nucleoplasmic lamin A/C signal. Interestingly, work by Ohad Medalia (Zwerger et al. J Cell Sci 2015) had previously shown that lamins A/C at the nuclear periphery appears to be mostly responsible for nuclear deformability (see Fig. 5 of the manuscript, where the authors used different DARPins to control nuclear lamin A/C localization to the nuclear periphery or interior). This would should be cited by the authors.

In Suppl. Fig. S1A, the authors show the effect of serum starvation on cell cycle distribution, with the expected increase of cells in G0/G1 phase. The reviewer is confused, however, how the authors can obtain such a smooth histogram with only 40 data points (based on the information provided in the response to reviewers), suggesting that the authors fit a curve to the data points. Without showing the actual data points, it is not possible to assess how good and robust the fit is, thus making the data difficult to interpret. Furthermore, the corresponding figure legend ("Narrow peak in serum starved cells indicate that cells are in the same cell cycle phase compared to a broader peak in cells grown in serum containing media") is confusing, as the data is demonstrating that the serum-starvation increases the fraction of cells in G0/G1.

The authors presented several data in the response to reviewers that are not included in the revised manuscript. For example, the comparisons between isolated nuclei and intact cells for the change in volume, bulk modulus, and anisotropic deformation provide important context and is very helpful for the interpretation of the data. As the authors acknowledge in their response, the absolute values of the bulk modulus pf isolated nuclei are slightly different than those measured in intact cells, potentially reflecting effects of the cytoskeleton. Inclusion of these data does not take away from the authors findings, but instead strengthens their overall conclusions and provides additional data to the scientific community. Thus, these data should be included in the manuscript.

The authors state in their response regarding the comments about the mCherry-lamin A overexpression and lamin A/C depletion that "All cells have been clearly identified as to how their lamin expression was modified as per the reviewer's suggestion". Nonetheless, none of the results (neither in the text nor in the figures) indicate in which experiments these cells were used. Thus, it remains unclear whether the results presented throughout the manuscript represent non-modified cells with heterogeneous lamin A/C expression levels, or include cells with lamin A/C depletion or knockdown. Since overexpression and depletion experiments would require mock or non-target controls, respectively, providing this information is critical for the interpretation of the experiments. If the authors did not include results from cell with mCherry-lamin A overexpression or lamin A/C knockdown cells, the methods section should be revised accordingly to eliminate the description.

The authors state in the figure legend for Figure 4 that outliers were ignored for the statistical analysis. To ensure robust results, it is important to clearly state how data points were identified as outliers, and how many data points were excluded as outliers.

One of the conclusions by the authors' is that cells with high levels of lamin A/C have increased variance in the lamin A/C levels. The authors should discuss to what extent low signal-to-noise levels and thus detection efficiency in cells with low levels of lamin A/C may contribute to these results.

© 2021. Published by The Company of Biologists under the terms of the Creative Commons Attribution License (https://creativecommons.org/licenses/by/4.0/).

Page 5, lines 97-98. The authors state “NIH 3T3 cells were obtained from America Type Culture Collection (ATCC) and marked free from any contamination”, but the authors provide no details how the authenticity of cell lines was validated and how lack of contamination was determined, e.g. whether the authors used STR profiling of the cells used in the experiments to rule out potential contamination with other cell lines in the author’s laboratory.

The authors state that cells were stably transfected with GFP-tagged lamin A/C chromobody. This implies that the authors must have performed some selection to only keep cells with stable expression of the construct, but no details are provided for this approach.

The manuscript still contains numerous instances where the authors refer to lamin A, instead of lamin A/C, e.g. in line 47, 51, 53, etc.

Reviewer 3

Advance summary and potential significance to field

The paper from Srivastava et al. provides a novel quantitative description of the well-known relationship between lamin A/C and nuclear stiffness. The authors tested the bulk compressibility of 3T3 NHI fibroblasts nuclei by exposing them to varying osmotic pressure, thus confirming that nuclear compression increased in low lamin A/C cells. Furthermore, the authors provided qualitative and quantitative details about the anisotropy of nuclei upon compressive stress, as well as of the heterogeneous distribution of lamin A/C in the nuclear envelope.

Comments for the author

The article is well constructed, the experiments were properly conducted, and the analysis is clear. However, there are a few things that may greatly improve it.
Although nuclear anisotropy due to compressive stress is not a novel result the authors provide an interesting quantitative description of the deformation in three dimension, and of the relation of the three spatial axis with lamin A/C expression. However, the section describing the anisotropy of the compressible nuclei could be improved by adding in the text quantitative details displayed in figure 2.
Overall, the text in the results section is formulated in a way that sounds very qualitative. It would be preferable to add more quantitative details considering that the authors made it clear that the point of their paper is to investigate the quantitative effects of lamin A/C on nuclear mechanics.

Minor comment:
There are consistent typing mistakes where the references are included in the manuscript (space missing between last word in the sentence and the opening bracket of the reference).

Reviewer 4

Advance summary and potential significance to field

Potential advance in understanding lamin contributions to nuclear volume changes.

Comments for the author

Much is understood about nuclear mechanics and the contributions of lamin-A/C, but more insights are perhaps gained by considering volume changes. The first review and the rebuttal have a number of issues that need to be addressed plus a couple more.
1) The rebuttal states that "we were unable to find any report of the quantitative relationship between lamin A/C expression or distribution and nuclear stiffness." However, the cited paper Harada et al (2014) explicitly plot "Nuclear stiffness" as a function of GFP-lamin-A in A549 cells (with constant lamin-B), and they show a power law scaling (exponent of 0.5) that also fits the trend from other cell types (Fig.S4D).

© 2021. Published by The Company of Biologists under the terms of the Creative Commons Attribution License (https://creativecommons.org/licenses/by/4.0/).

2) Key comments of the Reviewer remain unaddressed: "it is possible that the observed effects reflect these indirect effects of lamin on chromatin organization. While the authors briefly discuss this, they do not include any experiments to address this question". Furthermore, the submission "does not adequately address the underlying molecular mechanisms, or alternative explanations."
3) Osmotic compression of the nucleus and the cells was previously quantified in terms of volume changes in Fig.4D of Irianto et al (Biophy J 2013), which show the same trends as Fig.1B,C of Srivastava et al. Irianto also provide clear evidence of chromatin compaction or condensation by EM and by quantitative fluorescence measurements. Moreover, Irianto et al also show nuclear height decreases greatly in cell monolayers whereas nuclear area does not change much; this is the same result as Fig.2B here. The authors include in their rebuttal some data that does not seem to be in the manuscript but needs to be: "the new figure to the right depicts the percent nuclear deformation in nuclei that have been removed from cells and isolated (n =10 cells, ...)." Moreover, some evidence is needed that (1) the residual cytoskeleton and other non-nuclear structures are lacking, and (2) the Z-axis is preserved upon isolation.
4) Srivastava et al's images of 'epitope masking' in the rebuttal are clear, but they do not address whether similar effects explain the circumferential variations of Fig.3,4. One possible approach is to image GFP-lamin-A/C in these cells.
5) For the particular cells studied, the physiological relevance of osmotic pressure changes needs to be explained.

Second revision

<u>Author response to reviewers' comments</u>

Reviewers' comments (black)
Author's comments (blue)

Reviewer 1 Advance Summary and Potential Significance to Field
The authors addressed all my questions and the manuscript reads well now.

Reviewer 1 Comments for the Author
no further comments

We thank the reviewer for their help with our manuscript.

Reviewer 2 Advance Summary and Potential Significance to Field
The authors have submitted a revised manuscript that addresses several of the previous reviewers' concerns. The authors have now included additional data to increase the robustness of their key findings. The central novelty of the manuscript is the single cell based analysis that shows a strong linear correlation between lamin A/C levels and the nuclear bulk modulus, and the correlation of local nuclear deformation and local lamin A/C levels. These results will be of interest to the cell biology community.

We are glad our work and revised manuscript have addressed the reviewer's principal concerns, and that they see the findings as valuable to the biology community.

Reviewer 2 Comments for the Author
Nonetheless, several concerns remain to be addressed by the authors to sufficiently support their conclusions and to ensure scientific rigor and reproducibility of the work. For most of their experiments, the authors now include data from larger numbers of cells, e.g. the data provided in Figures 1D, 1E, 2C, 3C, 4H, S1E, which is a substantial improvement. However, other conclusions are still based on an extremely small number of cells, such as the results presented in Figs. 1F, 3D, making their interpretation difficult. For example, based on the results presented in Fig. 1G, the authors postulate a different nuclear dynamic mechanical behavior for cells with a lamin A/C fluorescence value above 16.9, but this conclusions is based on the behavior of just two cells below that threshold, which is insufficient to make such a claim. The authors should either provide

© 2021. Published by The Company of Biologists under the terms of the Creative Commons Attribution License (https://creativecommons.org/licenses/by/4.0/).

additional data to support their conclusions, or revise their statements.

We observe apparent regimes of gradual or rapid compression based on low or high lamin A/C expression respectively; to bolster this distinction, we have added additional data (n=32). We noted that single exponential fits distinguish between the regimes, and plot 6 examples of this in Fig 1G. While the detailed time-dependent mechanical analysis is beyond the scope of this manuscript, we report the compression time-constant as a function of lamin A/C expression level, showing that with increasing expression, the time-constant generally decreases (new inset Fig 1H).

Furthermore, important information on the data used for the analysis is missing. The authors do not include the (required) information in the figure legends on how many times the experiments shown were replicated in the laboratory. Such statements are critical to assess how robust the findings are, given the large variability inherent to many cell biology experiments. The authors should ensure that all figure legends contain this information.

We have ensured that all figure legends clearly indicate not only the sample size, but the number of times each experiment was replicated in the laboratory.

The authors continue to state in their abstract and other sections of the text that “the quantitative relationship between lamin A/C and nuclear deformation is unknown”. This statement is not correct, as numerous groups have shown quantitative relationships between lamin expression and nuclear deformation (e.g. nuclear strain, nuclear deformation, or stiffness). In addition to the publications already pointed out in the previous reviewer’s comments, other examples includes work by Schaepe et al. (Biophys J 2009, Fig. 4) and Swift et al. (Science 2013, Fig. 7; note that although the figure plots the lamin A:B ratio in the x-axis, other experiments in the same manuscript showed that lamin B is essentially constant, and therefore the graph essentially represents a plot of the lamin A levels). The authors should revise their text to state that the relationship between the nuclear bulk modulus and lamin A/C levels had not been determined previously, and that they now provide a single cell (and subcellular cell) based analysis. This would provide a more accurate reflection of the novelty provided by this manuscript.

We must disagree with the reviewer. In our previous rebuttal, we described in detail how all of the prior works referenced by the reviewer do not quantitatively plot stiffness (or modulus or compliance) as a function of lamin A/C expression or its distribution.
As referenced by the reviewer: Schaepe et al. BJ 2009 Fig 4, plots stiffness as a function of injected DNA concentration [NOTE: We have removed a figure which was provided for the referees. It showed Fig. 4 from **Schaepe et al.** (2009) Influence of lamin A on the mechanical properties of amphibian oocyte nuclei measured by atomic force microscopy. *Biophysical Journal* **96(10)**, 4319-4325. (DOI: 10.1016/j.bpj.2009.02.048)]

The data here are quite sparse, do not appear to be statistically significant, and critically do not connect stiffness with lamin concentration, but only rather weakly with respect to lamin coding DNA concentration. To our knowledge, no validation of lamin expression as a function of DNA concentration in the study referenced was performed.

In Swift et al 2013, the reviewer states that in Fig 7 the stiffness is plotted vs the ratio of lamin A:B, but that lamin B is essentially constant based on other data from that paper, and so the plot is effectively that of stiffness vs lamin A. [NOTE: We have removed a figure which was provided for the referees. It showed Fig. 7D from **Swift et al.** (2013) Nuclear lamin-A scales with tissue stiffness and enhances matrix-directed differentiation. *Science*, **341(6149)**. (DOI: 10.1126/science.1240104)]
We do not agree that lamin B is shown to be completely constant in all circumstances in the manuscript. Nevertheless, even if it were, no quantitative analysis of stiffness vs lamin A/C is offered in the Swift paper. It is unlikely that a reader would make the leaps of assuming that lamin B is constant, and thus Fig7D is really a plot of stiffness vs Lamin A/C, to arrive at a qualitative conclusion that stiffness scales with lamin A/C (and independent of lamin B); however, even if a reader did so, no quantitative expression exists between stiffness and lamin A/C in the Swift paper, and so we believe that our statement “the quantitative relationship between lamin A/C and nuclear deformation is unknown” is accurate and has not previously been measured. This is not a shortcoming of prior work, but also not previously quantitatively shown.

© 2021. Published by The Company of Biologists under the terms of the Creative Commons Attribution License (https://creativecommons.org/licenses/by/4.0/).

In the images presented in the manuscript and in the corresponding analysis, the authors show lamin A/C (almost) to be located almost exclusively at the nuclear periphery, even though lamins also form stable structures in the nuclear interior (see for example work by Robert Goldman and Roland Foisner). The authors should comment on whether the lack of nucleoplasmic lamin A/C signal in their data is reflective of the specific cell line and maybe the selection of cells in G0/G1 phase, or whether the deconvolution and image processing contributed to the elimination of nucleoplasmic lamin A/C signal.

We see no significant stable nucleoplasmic lamin in our study. The lamin A/C localization is unlikely associated with cell type, and not a product of imaging. As lamin concentrations change during the cell cycle, we synchronize cells for measurement, and the observed lamin A/C periphery may reflect using cells in stable G0/G1 phases, as those in karyokinesis are likely to have highly dynamic and variable structure, making quantitative comparisons difficult.

Interestingly, work by Ohad Medalia (Zwerger et al. J Cell Sci 2015) had previously shown that lamins A/C at the nuclear periphery appears to be mostly responsible for nuclear deformability (see Fig. 5 of the manuscript, where the authors used different DARPins to control nuclear lamin A/C localization to the nuclear periphery or interior). This would should be cited by the authors.

We have included the suggested reference in our manuscript

In Suppl. Fig. S1A, the authors show the effect of serum starvation on cell cycle distribution, with the expected increase of cells in G0/G1 phase. The reviewer is confused, however, how the authors can obtain such a smooth histogram with only 40 data points (based on the information provided in the response to reviewers), suggesting that the authors fit a curve to the data points. Without showing the actual data points, it is not possible to assess how good and robust the fit is, thus making the data difficult to interpret. Furthermore, the corresponding figure legend ("Narrow peak in serum starved cells indicate that cells are in the same cell cycle phase compared to a broader peak in cells grown in serum containing media") is confusing, as the data is demonstrating that the serum-starvation increases the fraction of cells in G0/G1.

Our distribution narrowing reflects that synchronization, and similar such plots may be found in numerous prior works such as Gomes et al. (Cell Div 2018) and Fan et al. (Theranostics 2017). We have further increased the dataset to 130 cells in Supplementary Fig. 1A clearly showing cells are synchronized in G0/G1 phase after serum starvation and replaced the fit with histogram data bins.

The authors presented several data in the response to reviewers that are not included in the revised manuscript. For example, the comparisons between isolated nuclei and intact cells for the change in volume, bulk modulus, and anisotropic deformation provide important context and is very helpful for the interpretation of the data. As the authors acknowledge in their response, the absolute values of the bulk modulus pf isolated nuclei are slightly different than those measured in intact cells, potentially reflecting effects of the cytoskeleton. Inclusion of these data does not take away from the authors findings, but instead strengthens their overall conclusions and provides additional data to the scientific community. Thus, these data should be included in the manuscript.

We have added these in Supplemental Fig S2C

The authors state in their response regarding the comments about the mCherry-lamin A overexpression and lamin A/C depletion that "All cells have been clearly identified as to how their lamin expression was modified as per the reviewer's suggestion". Nonetheless, none of the results (neither in the text nor in the figures) indicate in which experiments these cells were used. Thus, it remains unclear whether the results presented throughout the manuscript represent non-modified cells with heterogeneous lamin A/C expression levels, or include cells with lamin A/C depletion or knockdown. Since overexpression and depletion experiments would require mock or non-target controls, respectively, providing this information is critical for the interpretation of the experiments. If the authors did not include results from cell with mCherry-lamin A overexpression or lamin A/C knockdown cells, the methods section should be revised accordingly to eliminate the description.

© 2021. Published by The Company of Biologists under the terms of the Creative Commons Attribution License (https://creativecommons.org/licenses/by/4.0/).

We have assigned varied red hues to distinguish the data points with under, wild type and overexpression of lamin A/C in Fig. 1D and 1E.

The authors state in the figure legend for Figure 4 that outliers were ignored for the statistical analysis. To ensure robust results, it is important to clearly state how data points were identified as outliers, and how many data points were excluded as outliers.

The outliers were identified by plotting the residual plot using OriginPro software. Based on the residual values, the outliers were identified respectively. The figure below shows an example of the outlier selection. The points with a standardized residual value higher than +2 or less than -2 are generally considered as outliers. Accordingly, the outliers were marked in the residual plot (left) in red and the respective outliers have been shown in the stress/deformation vs lamin A/C density plot (right). An average of 4.6%±2.3% points were identified as outliers. [NOTE: We have removed a figure which was provided for the referees in confidence.]

One of the conclusions by the authors' is that cells with high levels of lamin A/C have increased variance in the lamin A/C levels. The authors should discuss to what extent low signal-to-noise levels and thus detection efficiency in cells with low levels of lamin A/C may contribute to these results.

We discussed this in supplementary Fig 3B, where we performed controls comparing varied amplification gain, signal to noise, and changes in the detected images. In brief, our results show that this is not a product of detection efficiency or noise. Moreover, if detection efficiency were an underlying mechanism in the variance (i.e. low signal to noise), then as the signal decreased, we would expect the distribution to broaden due to more noise. We observe the opposite, that as the signal increases, the distribution broadens.

Page 5, lines 97-98. The authors state "NIH 3T3 cells were obtained from America Type Culture Collection (ATCC) and marked free from any contamination", but the authors provide no details how the authenticity of cell lines was validated and how lack of contamination was determined, e.g. whether the authors used STR profiling of the cells used in the experiments to rule out potential contamination with other cell lines in the author's laboratory.

Upon arrival, the cells were immediately cultured and propagated. No observation suggests contamination, and cells were cultured separately from other cultures.

The authors state that cells were stably transfected with GFP-tagged lamin A/C chromobody. This implies that the authors must have performed some selection to only keep cells with stable expression of the construct, but no details are provided for this approach.

The GFP-tagged lamin A/C chromobody were selected with G418 at a concentration of 1000μg/ml in complete DMEM, and this has been added to the methods.

The manuscript still contains numerous instances where the authors refer to lamin A, instead of lamin A/C, e.g. in line 47, 51, 53, etc.

All the instances of lamin A/C have been modified and carefully checked throughout the manuscript.

Reviewer 3 Advance Summary and Potential Significance to Field

The paper from Srivastava et al. provides a novel quantitative description of the well-known relationship between lamin A/C and nuclear stiffness. The authors tested the bulk compressibility of 3T3 NHI fibroblasts nuclei by exposing them to varying osmotic pressure, thus confirming that nuclear compression increased in low lamin A/C cells. Furthermore, the authors provided qualitative and quantitative details about the anisotropy of nuclei upon compressive stress, as well as of the heterogeneous distribution of lamin A/C in the nuclear envelope.

Reviewer 3 Comments for the Author

The article is well constructed, the experiments were properly conducted, and the analysis is

© 2021. Published by The Company of Biologists under the terms of the Creative Commons Attribution License (https://creativecommons.org/licenses/by/4.0/).

clear. However, there are a few things that may greatly improve it.

We appreciate that the reviewer finds our article well-constructed, with clear analysis and properly conducted experiments, and appreciate the comments for improvement.

Although nuclear anisotropy due to compressive stress is not a novel result, the authors provide an interesting quantitative description of the deformation in three dimension, and of the relation of the three spatial axis with lamin A/C expression. However, the section describing the anisotropy of the compressible nuclei could be improved by adding in the text quantitative details displayed in figure 2.

To examine the relationship between lamin A/C expression and deformation in separate axes, we have added a Fig. 2B which shows that deformation in z axis is more for a low lamin A/C expressing cells and less for a more lamin A/C expressing cells, while major and minor axes deformations show a weaker relationship with lamin A/C concentration. To do so, we calculated the mean deformation in z-axis which was found to be 3-4 times larger than in plane axes.The text has been modified indicating the quantitative relevance of the results (line #171).

Overall, the text in the results section is formulated in a way that sounds very qualitative. It would be preferable to add more quantitative details, considering that the authors made it clear that the point of their paper is to investigate the quantitative effects of lamin A/C on nuclear mechanics.

Line #159 has been modified to include the quantification that with unit increase in lamin A/C fluorescence the nucleus stiffens by 1%.
Line #171 has been modified depicting that the deformation in z-axis was 3 to 4 time more than in the plane axes.
Line #190 has been modified to show that with unit increase in lamin A/C fluorescence the heterogeneity increases by 2%.

Minor comment:
There are consistent typing mistakes where the references are included in the manuscript (space missing between last word in the sentence and the opening bracket of the reference).

This spacing has been reformatted.

Reviewer 4 Advance Summary and Potential Significance to Field
Potential advance in understanding lamin contributions to nuclear volume changes.

Reviewer 4 Comments for the Author
Much is understood about nuclear mechanics and the contributions of lamin-A/C, but more insights are perhaps gained by considering volume changes. The first review and the rebuttal have a number of issues that need to be addressed plus a couple more.

3) The rebuttal states that "we were unable to find any report of the quantitative relationship between lamin A/C expression or distribution and nuclear stiffness." However, the cited paper Harada et al (2014) explicitly plot "Nuclear stiffness" as a function of GFP- lamin-A in A549 cells (with constant lamin-B), and they show a power law scaling (exponent of 0.5) that also fits the trend from other cell types (Fig.S4D).

As described in our rebuttal to reviewer 2, we do not agree that the mechanics of the nucleus has been clearly quantified as a function of lamin A/C. The data in Harada is virtually identical to that reported in Swift 2013; We do not agree that lamin B is shown to be completely constant in all circumstances in the Harada manuscript. Nevertheless, even if it were, no quantitative analysis of stiffness vs lamin A/C is offered in the Harada paper, it is unlikely that a reader would make the leaps of assuming that lamin B is constant, and thus FigS4D is really a plot of stiffness vs Lamin A/C, to arrive at a qualitative conclusion that stiffness scales with lamin A/C (and independent of lamin B), but even if a reader did so, no quantitative expression exists between stiffness and lamin A/C in the Harada paper, and so we believe that our statement “the quantitative relationship between lamin A/C and nuclear deformation is unknown” is accurate and has not previously been

© 2021. Published by The Company of Biologists under the terms of the Creative Commons Attribution License (https://creativecommons.org/licenses/by/4.0/).

measured. This is not a shortcoming of prior work, but not previously quantitatively shown.

4) Key comments of the Reviewer remain unaddressed: "it is possible that the observed effects reflect these indirect effects of lamin on chromatin organization. While the authors briefly discuss this, they do not include any experiments to address this question". Furthermore, the submission "does not adequately address the underlying molecular mechanisms, or alternative explanations."

Chromatin experiments, as well as other cellular proteins, are beyond the scope of this study which focuses on lamin A/C. Regarding the underlying molecular mechanisms, we describe how lamin A/C is known to be a structural protein which confers mechanical strength to the nucleus, and we find that increasing the expression of that protein leads to a stiffer nucleus. While nonlinearities or super-saturated states of lamin A/C are imaginable, we do not observe such behavior and limit our discussion to the observed linear mechanics phenomena.

5) Osmotic compression of the nucleus and the cells was previously quantified in terms of volume changes in Fig.4D of Irianto et al (Biophy J 2013), which show the same trends as Fig.1B,C of Srivastava et al. Irianto also provide clear evidence of chromatin compaction or condensation by EM and by quantitative fluorescence measurements. Moreover, Irianto et al also show nuclear height decreases greatly in cell monolayers whereas nuclear area does not change much; this is the same result as Fig.2B here.
The authors include in their rebuttal some data that does not seem to be in the manuscript but needs to be: "the new figure to the right depicts the percent nuclear deformation in nuclei that have been removed from cells and isolated (n =10 cells, ...)."

We have included the percent nuclear deformation in isolated nuclei as supplemental figure S2C.

Moreover, some evidence is needed that:
(1) the residual cytoskeleton and other non-nuclear structures are lacking

While we cannot be certain that no trace proteins remain, we are confident that the intact cytoskeleton is removed. The bottom left image shows a nucleus inside the cell where actin cytoskeleton is stained with phalloidin (red) and the nucleus is stained with DAPI (blue); whereas the image on the right shows an isolated nucleus also stained with phalloidin but depicting the complete absence of actin filaments, confirming that isolated nucleus lacks actin cytoplasmic structure after isolation. [NOTE: We have removed a figure which was provided for the referees in confidence.]

(2) the Z-axis is preserved upon isolation.
The isolated nuclei upon settling and attaching to glass substrates appear to lay flat rather than stand on end. This would be expected with any high-aspect ratio object, and preserves the z axis as vertical from a 2D intact cell.

6) Srivastava et al's images of 'epitope masking' in the rebuttal are clear, but they do not address whether similar effects explain the circumferential variations of Fig.3,4. One possible approach is to image GFP-lamin-A/C in these cells.

The epitope masking on the basal surface of the nucleus mentioned by Ihalainen et al may be due to cell adhesion as the antibody has poor access to the regions adhered to the substrate. Whereas on the apical and medial circumferential planes there is unhindered access of epitopes to the antibodies resulting in non-epitope masking in “XY " planes as is the case in our study. This observation is clearly backed up by Ihalainen et al as the authors quantified lamin A/C fluorescence variation in apical vs basal planes imaged from the side “YZ" but illustrate homogeneity in “XY” planes. As they report no epitope masking in accessible regions, we do not suspect it here.

7) For the particular cells studied, the physiological relevance of osmotic pressure changes needs to be explained.

© 2021. Published by The Company of Biologists under the terms of the Creative Commons Attribution License (https://creativecommons.org/licenses/by/4.0/).

There is not necessarily any physiological relevance of the osmotic pressure changes, just like there is not necessarily physiological relevance of an AFM cantilever; osmotic pressure provides the dynamic range of stress that allows the measurement to be performed. We do not propose or preclude the role of osmotic stresses in any biological context.

Third decision letter

MS ID#: JOCES/2020/248559

MS TITLE: Spatial distribution of lamin A/C determines nuclear stiffness and stress-mediated deformation

AUTHORS: Luv Kishore Srivastava, Zhaoping Ju, Ajinkya Ghagre, and Allen J. Ehrlicher
ARTICLE TYPE: Short Report

We have now reached a decision on the above manuscript.

To see the reviewers' reports and a copy of this decision letter, please go to: https://submit-jcs.biologists.org and click on the 'Manuscripts with Decisions' queue in the Author Area. (Corresponding author only has access to reviews.)

As you will see, the reviewers find many aspects of the revisions compelling and do not have further requests for major experiments. However, all three reviewers have significant concerns about the accuracy and precision of certain statements in the text and also raise issues about the degree to which the current analysis is sufficiently quantitative and whether the quantitative rigor of the analyses are reflected in the corresponding descriptions in the text of the manuscript. I hope that you will be able to carry these out, because I believe these changes would significantly improve the rigor and impact of your manuscript. I would then be happy to return the manuscript to reviewers.

We are aware that you may be experiencing disruption to the normal running of your lab that makes experimental revisions challenging. If it would be helpful, we encourage you to contact us to discuss your revision in greater detail. Please send us a point-by-point response indicating where you are able to address concerns raised (either experimentally or by changes to the text) and where you will not be able to do so within the normal timeframe of a revision. We will then provide further guidance. Please also note that we are happy to extend revision timeframes as necessary.

Please ensure that you clearly highlight all changes made in the revised manuscript. Please avoid using 'Tracked changes' in Word files as these are lost in PDF conversion.

I should be grateful if you would also provide a point-by-point response detailing how you have dealt with the points raised by the reviewers in the 'Response to Reviewers' box. Please attend to all of the reviewers' comments. If you do not agree with any of their criticisms or suggestions please explain clearly why this is so.

Reviewer 2

Advance summary and potential significance to field

The authors have submitted a revised manuscript that includes additional experimental data and detail that address the main concerns of the reviewersÂ’. Nonetheless, a few minor concerns remain that should be addressed by the authors.

© 2021. Published by The Company of Biologists under the terms of the Creative Commons Attribution License (https://creativecommons.org/licenses/by/4.0/).

Comments for the author

Exclusion of outliers (Figure 4H) is problematic and may hide potentially interesting biological findings. The lamin A/C density vs. stress/deformation plot shown in the response to the reviewers reveals a distinct cluster of cells with low lamin A/C levels and yet high stress/deformation values. Comparing the figure in the response to the reviewers with Figure 4F suggests that these data points are actually included in the graph, alleviating concerns. However, since the figure legend indicates that outliers have been excluded for the correlation analysis (Fig. 4H), the authors should clearly mark the excluded outliers in the figure and include a description in the methods section how the outliers were identified, similar to what was done in the response to reviewers.

The response to reviewer #4 regarding epitope masking as a potential explanation for variations in lamin A/C intensities suggests that the reduced labeling of the basal nuclear surface is due to limited antibody access due to the cell substrate. However, the Ihalainen et al. manuscript shows that lamin B1 does not show the same reduction in basal labeling, making this explanation unlikely to hold up. Thus, the authors cannot rule out epitope masking as a potential source of variation and should acknowledge this possibility in the manuscript.

Despite the authors' argumentation, the statement in the abstract that "the quantitative relationship between lamin A/C and nuclear deformation is unknown" is overly broad and factually incorrect. In their response, the authors argue that previous studies do not directly linking lamin levels to nuclear stiffness, elastic modulus, or compliance. However, previous studies HAVE shown quantitative measurements of nuclear deformation (i.e. strain, normalized deformation) as a function of lamin levels. The authors' statement in the abstract explicitly refers to "nuclear deformation", which the authors acknowledge in their own response as having been described previously. Aside from the discussion whether previous studies plotted deformation or inferred nuclear mechanical properties from these plots, there is little doubt that extensive research has been done how changes in lamin levels quantitatively alter nuclear deformation. I do not want to take away any novelty of the authors, but dismissing 15-plus years of research by numerous groups as not having produced any quantitative relationship between lamin A/C and nuclear deformation is just not accurate. Thus, this statement in the abstract should be rephrased accordingly.

Discussion, page 11, line 237. The authors write that pathological conditions are "related to a mutation in the lmna gene…". Please note that more than 400 mutations have been identified to date that cause a variety of diseases. Thus, the statement should be revised to "…related to mutations in the LMNA gene",

Reviewer 3

Advance summary and potential significance to field

The paper from Srivastava et al. provides a novel quantitative description of the well-known relationship between lamin A/C and nuclear stiffness. The authors tested the bulk compressibility of 3T3 NHI fibroblasts nuclei by exposing them to varying osmotic pressure, thus confirming that nuclear compression increased in low lamin A/C cells. Furthermore, the authors provided qualitative and quantitative details about the anisotropy of nuclei upon compressive stress, as well as of the heterogeneous distribution of lamin A/C in the nuclear envelope.

Comments for the author

The article is well constructed, the experiments were properly conducted, and the analysis is clear. However, there are a few things that may greatly improve it.
Although nuclear anisotropy due to compressive stress is not a novel result the authors provide an interesting quantitative description of the deformation in three dimension, and of the relation of the three spatial axis with lamin A/C expression. However, the section describing the anisotropy of the compressible nuclei could be improved by adding in the text quantitative details displayed in figure 2.

© 2021. Published by The Company of Biologists under the terms of the Creative Commons Attribution License (https://creativecommons.org/licenses/by/4.0/).

Overall, the text in the results section is formulated in a way that sounds very qualitative. It would be preferable to add more quantitative details considering that the authors made it clear that the point of their paper is to investigate the quantitative effects of lamin A/C on nuclear mechanics.

Minor comment:
There are consistent typing mistakes where the references are included in the manuscript (space missing between last word in the sentence and the opening bracket of the reference).

Reviewer 4

Advance summary and potential significance to field

Use of osmolarity to measure bulk modulus of nucleus versus lamin A/C.

Comments for the author

The authors should be much more responsive and forthcoming in modifying their text to address all of the concerns of the various expert reviewers in order to make this submission a more scholarly contribution to the field. In other words, most comments in the previous review should be explicitly reflected in some change to the text and/or figures.
Further explanation is also needed on the following points:

1. The authors must add to the main text the sentence: "Based on comparisons to antibody in 40 cells, a mid-range chromobody signal (50+/-10 on scale of 0-100) corresponds to a 2-fold range of antibody fluorescence. Also, for cells with the lowest chromobody signal, antibody fluorescence is non-zero and about half the mid-range intensity of antibody, which indicates limited sensitivity of chromobody to low levels of lamin A/C." This is because their Supp Fig.S1c compares chromobody fluorescence in 40 cells to what they say is "antibody staining (Atto-647N Sigma) as a gold standard", showing cells with a relatively narrow mid-range antibody signal of 60-70 corresponds to a very broad chromobody signal of 25-80. Conversely, a mid-range chromobody signal of 40-60 corresponds to an antibody signal of 55-110.
In the section "Quantification of lamin A/C expression using antibodies and lamin A/C chromobody". The authors must further discuss in the main text the caveats on the transfection approach (which Reviewer 2 rightly asked more details about):
"As with any transfection, the level of GFP tagged lamin A/C chromobody will differ between cells because some cells receive more plasmid than other cells."

2. The Abstract must be changed from 'The nuclear membrane protein, lamin A/C, is known to be a dominant component in nuclear stiffening; however, the quantitative relationship between lamin A/C and nuclear deformation is unknown.' to "The nuclear membrane protein, lamin A/C, is known to be an important contributor to nuclear stiffening, but bulk moduli of nuclei have not been reported for various levels of lamin A/C."

3. The Abstract must be made more accurate and specific: "Here we measure the nuclear bulk moduli as a function of lamin A/C expression and applied osmotic stress, revealing a linear dependence within the range of 2-4 MegaPascal (MPa)."

4. The authors must also include somewhere in their text what was pointed out previously:
Harada et al (2014) plot "Nuclear stiffness" as a function of varied expression of GFP-lamin-A in A549 cells using micropipette aspiration (Fig.S4D), and for the A549 cells, they show (in Fig.1C) that lamin-B1,2 does not vary by more than ~10% between cells and does not change with siLMNA in these cells (Fig.1F). Importantly, they show power law scaling (exponent of 0.5 in Fig.S4D) for stiffness with varying intensity of GFP-lamin-A, and this power law also fits the trend from other cell types with different levels of lamin-B.

© 2021. Published by The Company of Biologists under the terms of the Creative Commons Attribution License (https://creativecommons.org/licenses/by/4.0/).

Third revision

Author response to reviewers' comments

Reviewer (Black)
Authors (Blue)

Reviewer 2 Advance Summary and Potential Significance to Field:

The authors have submitted a revised manuscript that includes additional experimental data and detail that address the main concerns of the reviewers'. Nonetheless, a few minor concerns remain that should be addressed by the authors.

Reviewer 2 Comments for the Author:

Exclusion of outliers (Figure 4H) is problematic and may hide potentially interesting biological findings. The lamin A/C density vs. stress/deformation plot shown in the response to the reviewers reveals a distinct cluster of cells with low lamin A/C levels and yet high stress/deformation values. Comparing the figure in the response to the reviewers with Figure 4F suggests that these data points are actually included in the graph, alleviating concerns. However, since the figure legend indicates that outliers have been excluded for the correlation analysis (Fig. 4H), the authors should clearly mark the excluded outliers in the figure and include a description in the methods section how the outliers were identified, similar to what was done in the response to reviewers.

As requested by the reviewer, the outliers have been clearly marked in Figure 4F (in red) and the description of the identification process has been added in line # 160 of the manuscript.

The response to reviewer #4 regarding epitope masking as a potential explanation for variations in lamin A/C intensities suggests that the reduced labeling of the basal nuclear surface is due to limited antibody access due to the cell substrate. However, the Ihalainen et al. manuscript shows that lamin B1 does not show the same reduction in basal labeling, making this explanation unlikely to hold up. Thus, the authors cannot rule out epitope masking as a potential source of variation and should acknowledge this possibility in the manuscript.

We postulated a hypothesis which could have been a possible reason for epitope masking, and we agree with the reviewer that if lamin B1 does not show any epitope masking, then probably there is a different mechanism behind this observation. While the manuscript by Ihalainen et al. has clearly shown that epitope masking was found on the basal surface of the nucleus which brings xz plane into account and neither they nor have any previous research to our knowledge observed masking in the xy plane. All the lamin A/C fluorescence quantification in our manuscript has been done in xy plane which we believe eliminates the possibility of epitope masking. Nevertheless, as requested by the reviewer, we have mentioned this as a potential cause for antibody labeling variation and cited Ihalainen in line # 113.

Despite the authors' argumentation, the statement in the abstract that "the quantitative relationship between lamin A/C and nuclear deformation is unknown" is overly broad and factually incorrect. In their response, the authors argue that previous studies do not directly linking lamin levels to nuclear stiffness, elastic modulus, or compliance. However, previous studies HAVE shown quantitative measurements of nuclear deformation (i.e. strain, normalized deformation) as a function of lamin levels. The authors' statement in the abstract explicitly refers to "nuclear deformation", which the authors acknowledge in their own response as having been described previously. Aside from the discussion whether previous studies plotted deformation or inferred nuclear mechanical properties from these plots, there is little doubt that extensive research has been done how changes in lamin levels quantitatively alter nuclear deformation. I do not want to take away any novelty of the authors, but dismissing 15-plus years of research by numerous groups as not having produced any quantitative relationship between lamin A/C and nuclear deformation is just not accurate.
Thus, this statement in the abstract should be rephrased accordingly.

© 2021. Published by The Company of Biologists under the terms of the Creative Commons Attribution License (https://creativecommons.org/licenses/by/4.0/).

We regret the confusion this minor word choice has produced, and in interest of resolving have modified the abstract and the modified statement in line #14 is “Though the nuclear membrane protein lamin A/C is known to contribute to nuclear stiffness, bulk moduli of nuclei have not been reported for various levels of lamin A/C. Here we measure the nuclear bulk moduli as a function of lamin A/C expression and applied osmotic stress, revealing a linear dependence within the range of 2-4 MegaPascal (MPa).”

Discussion, page 11, line 237. The authors write that pathological conditions are “related to a mutation in the lmna gene…”. Please note that more than 400 mutations have been identified to date that cause a variety of diseases. Thus, the statement should be revised to “…related to mutations in the LMNA gene”,

As per the reviewer’s suggestion we have modified the text from “a mutation” to “mutations” in line #256

Reviewer 4 Advance Summary and Potential Significance to Field:

Use of osmolarity to measure bulk modulus of nucleus versus lamin A/C.

Reviewer 4 Comments for the Author:

The authors should be much more responsive and forthcoming in modifying their text to address all of the concerns of the various expert reviewers in order to make this submission a more scholarly contribution to the field. In other words, most comments in the previous review should be explicitly reflected in some change to the text and/or figures. Further explanation is also needed on the following points:

We thank the reviewer for sharing their perspective with us.

8) The authors must add to the main text the sentence: "Based on comparisons to antibody in 40 cells, a mid-range chromobody signal (50+/-10 on scale of 0-100) corresponds to a 2-fold range of antibody fluorescence. Also, for cells with the lowest chromobody signal, antibody fluorescence is non-zero and about half the mid-range intensity of antibody, which indicates limited sensitivity of chromobody to low levels of lamin A/C." This is because their Supp Fig.S1c compares chromobody fluorescence in 40 cells to what they say is "antibody staining (Atto-647N Sigma) as a gold standard", showing cells with a relatively narrow mid-range antibody signal of 60-70 corresponds to a very broad chromobody signal of 25-80. Conversely, a mid-range chromobody signal of 40-60 corresponds to an antibody signal of 55-110. In the section "Quantification of lamin A/C expression using antibodies and lamin A/C chromobody". The authors must further discuss in the main text the caveats on the transfection approach (which Reviewer 2 rightly asked more details about): "As with any transfection, the level of GFP tagged lamin A/C chromobody will differ between cells because some cells receive more plasmid than other cells."

We believe that the suggested text:

"Based on comparisons to antibody in 40 cells, a mid-range chromobody signal (50+/-10 on scale of 0-100) corresponds to a 2-fold range of antibody fluorescence. Also, for cells with the lowest chromobody signal, antibody fluorescence is non-zero and about half the mid-range intensity of antibody, which indicates limited sensitivity of chromobody to low levels of lamin A/C."

is vague and confusing, and although the reviewer is adamant, we humbly suggest that perhaps reporting the Pearson’s correlation value offers a more statistical and conventional description of the data.

To that end we have added, “Based on comparisons between chromobody fluorescence and antibody labeling fluorescence in 40 cells, we compute a linear fit with Pearson’s r value of 0.75”

Rather than :” antibody fluorescence is non-zero and about half the mid-range intensity of antibody, which indicates limited sensitivity of chromobody to low levels of lamin A/C." Directly

© 2021. Published by The Company of Biologists under the terms of the Creative Commons Attribution License (https://creativecommons.org/licenses/by/4.0/).

following the chromobody fit discussion, we have added: “While chromobody and antibody fluorescence have a linear relationship over the majority of the observed range, the Y intercept of their fit is negative, suggesting that the antibody has greater sensitivity than the chromobody in detecting very low expression levels of lamin A/C.”

Regarding the GFP tagged lamin A/C chromobody, the reviewer is correct that the transfection level of chromobody can influence its fluorescence intensity. We have thus modified the reviewer’s suggested text: "As with any transfection, the level of GFP tagged lamin A/C chromobody will differ between cells because transfection efficiency essentially depends on factors like cell density, transfection time, phase of cell cycle, batch of plasmid DNA and DNA concentration." to #109. However, to minimize the variation in transfection level, the above-mentioned factors were kept the same in all instances of transfection. The chromobodies once expressed bind with the lamin A/C and are mainly localized at the nuclear membrane. Hence, two cells with similar chromobody transfection level varying in their lamin A/C expression, would have distinct spatial fluorescence. Thus, provided that the chromobody expression level exceeds that of lamin A/C, the localized chromobody fluorescence linearly matches that of lamin A/C. Without this relationship, our analysis in Fig S1c-f would not be possible.

9) The Abstract must be changed from 'The nuclear membrane protein, lamin A/C, is known to be a dominant component in nuclear stiffening; however, the quantitative relationship between lamin A/C and nuclear deformation is unknown.' to "The nuclear membrane protein, lamin A/C, is known to be an important contributor to nuclear stiffening, but bulk moduli of nuclei have not been reported for various levels of lamin A/C."

Line #14 has been modified to “Though the nuclear membrane protein lamin A/C is known to contribute to nuclear stiffness, bulk moduli of nuclei have not been reported for various levels of lamin A/C.”

10) The Abstract must be made more accurate and specific: "Here we measure the nuclear bulk moduli as a function of lamin A/C expression and applied osmotic stress, revealing a linear dependence within the range of 2-4 MegaPascal (MPa)."

Line #15 has been modified to “Here we measure the nuclear bulk moduli as a function of lamin A/C expression and applied osmotic stress, revealing a linear dependence within the range of 2-4 MegaPascal (MPa).”

11) The authors must also include somewhere in their text what was pointed out previously: Harada et al (2014) plot "Nuclear stiffness" as a function of varied expression of GFP-lamin-A in A549 cells using micropipette aspiration (Fig.S4D), and for the A549 cells, they show (in Fig.1C) that lamin-B1,2 does not vary by more than ~10% between cells and does not change with siLMNA in these cells (Fig.1F). Importantly, they show power law scaling (exponent of 0.5 in Fig.S4D) for stiffness with varying intensity of GFP-lamin-A, and this power law also fits the trend from other cell types with different levels of lamin-B.

Line #70 has been modified to “Harada et al. showed that in cancerous solid tissue cells and mesenchymal stem cells, the apparent nuclear stiffness increases with relative increasing of lamin A to lamin B with a power law coefficient of 0.5, where lamin B levels vary little, suggesting that specifically lamin A expression levels play a key role in the nuclear elastic moduli. However, the quantitative scaling of nuclear bulk modulus as a function of lamin A/C expression remains unknown” in the manuscript.

Fourth decision letter

MS ID#: JOCES/2020/248559

© 2021. Published by The Company of Biologists under the terms of the Creative Commons Attribution License (https://creativecommons.org/licenses/by/4.0/).

MS TITLE: Spatial distribution of lamin A/C determines nuclear stiffness and stress-mediated deformation

AUTHORS: Luv Kishore Srivastava, Zhaoping Ju, Ajinkya Ghagre, and Allen J. Ehrlicher
ARTICLE TYPE: Short Report

I am happy to tell you that your manuscript has been accepted for publication in Journal of Cell Science, pending standard ethics checks.

Reviewer 2

Advance summary and potential significance to field

The authors have submitted a revised manuscript that addresses all of the previous concerns raised by the reviewers. The revised text provides a more accurate and nuanced description of the work, which will be of value to the cell biology community. Thus, I recommend publication of this work in Journal of Cell Science.

Comments for the author

No further suggestions. The authors have addressed all of the reviewers' comments.

Reviewer 3

Advance summary and potential significance to field

Quantitative description of the relationship between lamin A/C and nuclear stiffness.

Comments for the author

The quality of the paper has improved since the last submission, as the authors have addressed all concerns raised by the reviewers and modified the text according to all comments given.

Reviewer 4

Advance summary and potential significance to field

Please see previous comments.

Comments for the author

The authors were reasonably responsive to my comments.

© 2021. Published by The Company of Biologists under the terms of the Creative Commons Attribution License (https://creativecommons.org/licenses/by/4.0/).
